# Patterns of Inhalant Use among Incarcerated Youth

**DOI:** 10.1371/journal.pone.0135303

**Published:** 2015-09-02

**Authors:** Susan M. Snyder, Matthew O. Howard

**Affiliations:** 1 School of Social Work, Georgia State University, Atlanta, GA, United States of America; 2 School of Social Work, University of North Carolina at Chapel Hill, Chapel Hill, NC, United States of America; University of Ariel, ISRAEL

## Abstract

Inhalant use is especially prevalent among antisocial youth and can have serious health consequences. However, the extant literature has not investigated how use of various inhalants may co-occur among incarcerated youth. This study begins to address this gap in the literature by using latent class analyses to form distinct typologies of inhalant use. Study participants were residents (N = 723) of 27 Missouri Division of Youth Services facilities. Interviews assessed psychiatric symptoms, antisocial traits, delinquency, trauma, suicidality, and substance use behaviors. The mean age of the mostly male, ethnically diverse sample was 15.5 (S.D. = 1.2) years old. The study revealed the following classes of inhalant use: (1) severe polyinhalant use; (2) moderate polyinhalant use; (3) gas and permanent marker use; and (4) low-use. Compared to the low-use class, members of the severe polyinhalant use class had experienced more than double the rate of head injuries, the highest rates of traumatic experiences, and the highest rates of mental illness diagnoses. The gas and markers class had the highest rate of reporting hearing voices, followed by the severe polyinhalant use class, and the moderate polyinhalant use class. Results of this study underscore the need to address the high rate of head injuries and mental health diagnoses that contribute to severe polyinhalant use.

## Introduction

Adolescent inhalant use is a serious public health problem that disproportionally affects antisocial youth. The prevalence rates for antisocial youth approach 40% [1, 2], compared to nearly 8.8% of youth in the general population [3]. Despite the pernicious nature of inhalant use, studies have yet to explore typologies of inhalant use among incarcerated youth to better understand how use of various inhalants may co-occur. As a result, it is unclear what individual characteristics (i.e., demographic characteristics, personality traits, health conditions, mental health conditions or substance use behaviors) are associated with the most severe polyinhalant use. Here polyinhalant use refers to using an assortment of inhalants over a period of time; the use can be simultaneous or successive.

The National Institute on Drug Abuse [4] defines inhalants as “volatile substances that produce chemical vapors that can be inhaled to induce a psychoactive, or mind-altering effect” (p. 1). Examples of inhalants include glue, paint, gasoline, solvents, whipped cream dispensers, and nail polish remover [3–5]. Inhalant users “sniff,” “huff,” or “snort” fumes from containers, paper or plastic bags, soaked rags, or directly from aerosol cans. Effects of inhalant intoxication last only a few minutes and similar to alcohol, include slurred speech, ataxia, euphoria, and dizziness. Inhalant use can have deleterious health consequences including brain damage, heart irregularities, optic nerve damage, hearing loss, liver damage, muscle atrophy, and death [4–6].

Population-based studies have found that inhalant use is associated with mental illnesses [7], criminal activity [7–9], and use of multiple drugs [9, 10]. Using data from 68,126 adults who participated in the 2002 and 2003 National Surveys on Drug Use and Health, Wu and Ringwalt [9] found that 50% of adults who had used four or more classes of drugs had used inhalants during their lifetime. This study also found that lifetime inhalant users were more likely to be male, to have a higher level of family income, and to reside in metropolitan areas compared to non-users. Wu and Howard [7] used data from 43,093 participants in the 2001–2002 National Epidemiologic Survey on Alcohol and Related Conditions to explore the prevalence of psychiatric disorders among lifetime inhalant users. Wu and Howard found that 32% of lifetime inhalant users met the criteria for antisocial personality disorder.

A small body of literature examines inhalant use among antisocial youth. Howard and Jenson’s [1] study of 475 probationers in Utah found that 34.3% had used inhalants in their lifetime. This study also found that compared to non-users, inhalant users had significantly higher rates of suicidality, including ideation (52.1% vs. 32.2% respectively) and attempts (25.8% vs. 12.5% respectively). These findings suggest that inhalant use may be used as a form of self-medication for individuals who experience mental illnesses. Studies that have focused specifically on incarcerated youth have found that compared to non-users, inhalant users were significantly more likely to have been diagnosed with a mental illness, to have reported hearing voices of people who were not there [2], to have suicidal ideation or attempts [2, 11], to have experienced trauma, and to be impulsive [10].

Another key finding among incarcerated youth is that inhalant users were significantly more likely than non-users to have experienced a head injury with loss of consciousness [2, 11], also referred to as a traumatic brain injury (TBI) [12, 13]. The existing literature suggests that TBI inhibits healthy neurodevelopment in regions of the brain that govern judgment, impulsivity and addiction. Studies have linked TBI with the following cognitive impairments: memory, attention, decision-making, and slower cognitive processing speed. TBI is also associated with emotional lability, apathy, and impulsivity [14]. Newcombe et al.’s [15] study, which used MRIs and acquired diffusion tensor magnetic resonance imaging on 44 patients with TBI and 40 controls, found that TBI patients’ slowed perceptual and motor processing abilities were correlated with the diffusion coefficient in many areas of the brain, in particular the frontal cortices. The frontal cortices are responsible for inhibition, attention, planning and self-control. Catroppa et al. [16] found significantly lower IQs among youth with TBI compared to controls without TBI. Perhaps not surprisingly, youth with TBI are more likely to use substances, engage in delinquent behaviors, and be incarcerated [12, 17].

### Current Study

Patterns of polyinhalant use remain understudied. The extant literature has tended to focus on either a single inhalant, or has aggregated inhalants into a scale. Neither approach elucidates which youth engage in the most risky inhalant use behaviors. Because even using a single inhalant can result in serious physical consequences, including death [4], we are interested in youth who have used multiple inhalants because this behavior may be especially dangerous. As the first systematic effort to explore polyinhalant use among incarcerated youth, our study had the following two aims: (a) to determine typologies of polyinhalant use; (b) to explore differences among the groups based on demographic, psychiatric, personality, and substance use characteristics. The overall goal of this study is to further our understanding of the co-occurring use of specific inhalants, which could inform prevention and intervention strategies.

## Materials and Methods

### Study Sample

The Missouri Division of Youth Services (DYS) provides residential rehabilitation services at 27 statewide facilities. Facilities range from 8 to 102 beds. When Missouri’s juvenile courts commit 13–17 years old to care, DYS serves as these youths’ legal guardian. DYS youth are representative of a national sample of incarcerated youth based on age, sex, and the number of state youth incarcerated per 100,000 adolescents [18]. The research team targeted 4 DYS regions and the residential facilities within those regions for interviewing in sequential order. That is, all youth at one facility were interviewed, then our interviewing team moved to the next facility and interviewed all youth at that facility, until all such facilities and youth were interviewed. Using this approach, all facility residents were recruited for participation and no youth were re-interviewed at another facility. Over a 3-month period in 2004 one-session interviews lasting 30–90 minutes were completed. Interview length depended primarily on the respondent’s inhalant use history. If youth became fatigued during the interview they could take short breaks, but consistent with DYS policy the youth were always observed by a project interviewer. Although 740 current DYS residents were eligible to participate in the study, 10 youth were on furlough and 2 youth were transferred to another facility before they could be interviewed. All of the 728 available youth agreed to participate, but 5 interviews were discontinued because 4 youth displayed signs or reported symptoms of psychosis and one youth decided to terminate the interview. At the time interviews were conducted, 97.7% of DYS residents (N = 723) completed the interviews, which constituted 99.3% of residents available for interviewing, and approximately 55.0% of youth committed to DYS care in the previous year. The current study is a virtual census of the DYS population at the time the study was undertaken and also largely represents DYS annual residents.

Fifteen graduate social work student interviewers conducted the interviews with 530 (73.3%) interviews being conducted by 7 core interviewers. To reduce interviewer errors interviewers were required to complete an intensive 1-day training session. Each facility had an interview editor on-site as youth were interviewed; the interview editor inspected schedules, which minimized interviewer omissions and errors. Training entailed four hours of education regarding inhalant use, including types of inhalants used, health and social consequences of inhalant use, modes of inhalant use, approaches to the assessment of inhalant use, and general mentoring regarding clinical research interviewing in correctional settings (e.g., maintaining safety and confidentiality). The last four hours of the training consisted of a methodological review of the screening and interview assessments to ensure that interviewers were clear about the intent of all questions and how they should be phrased and recorded. Interviewers then completed two full interviews with mock interviewees so they could practice the interview in its entirety.

Each facility provided large rooms with private areas where confidential one-on-one interviews could be conducted. Prior to each interview, interviewers ensured that the interviews could not be overheard. The informed assent form and interview protocol informed residents about the study; the forms provided the name and telephone number for a non-study or university affiliated advocate to call for additional information about the study (DYS allowed youth to use telephones for this purpose at any time during business hours), assured youth that participation was not required, assured youth they could stop participating in the interview at any point, and advised youth that their participation or nonparticipation in the study would not affect their legal status in any way. Youth signed informed assent forms and received $10.00 in their facility monetary accounts (and a receipt confirming the deposit) for completing the interview. As the legal guardian of all incarcerated youth, DYS formally provided permission for youths’ study participation. The Missouri DYS IRB and the Washington University Human Studies Committee IRB (operating in strict accordance with the governing regulations for research on prisoners) approved informed consent and study protocols. The federal Office of Human Research Protections officially certified the project, and the National Institute on Drug Abuse granted the project a Certificate of Confidentiality. Privacy rights were thoroughly described for each participant and all participants received a copy of a Washington University brochure, “Your Privacy Matters…” and a copy of the informed assent agreement.

The interviews were not audio or video recorded. Respondent’s answers were recorded on hard copy questionnaires that did not include any personally identifying information for respondents. Once data entry of the questionnaire responses was completed, all questionnaires were shredded.

### Materials and Measures

#### Volatile Solvent Screening Inventory (VSSI)

The Volatile Solvent Screening Inventory (VSSI) is an approximately 45-min interview that captures demographic characteristics, medical history, lifetime and annual use of 65 inhalants, other drug use and substance-related problems, current psychiatric symptoms, suicidal ideation and actual suicide attempts, trauma history, antisocial traits and criminal activity [2]. This is the first study to use the VSSI, so reliability and validity data are unavailable for this measure.

Although respondents were asked about their lifetime use of 65 inhalants, this study included only the 16 variables with at least 5% of the sample saying “yes” to use of that inhalant. Helium was excluded from analyses it is not believed to cause intoxication and not classified as a psychoactive agent [19], and paint thinner was excluded because it was highly correlated with spray paint. Thus, analyses included the following 14 inhalants: airplane or model glue, anesthetic gases, Freon, gas from whipping cream cans, butane, "White-out" or another correction fluid, air freshener, nail polish, nail polish remover, whippets, spray paint, gas from computer "duster" sprays, permanent markers and gasoline. Youth were asked for each inhalant whether they had “ever inhaled or “huffed” [inhalant] through your nose or mouth in an effort to get high?” The majority of respondents were familiar with the term “huffing” and most appeared to understand the meaning of the inhalant use questions.

#### Demographic factors

Age, sex, self-reported racial or ethnic identity, geographical area of family residence (i.e., urban, suburban, small town, rural), and family receipt of public assistance were recorded for each youth.

#### Medical history

Dichotomous (yes = 1) questions asked if youth have ever had (1) a head injury that caused them to blackout for more than 20 minutes, (2) a psychiatrist or other doctor diagnosed them with a mental disorder, (3) they “heard voices of people who were not actually there.”

#### Delinquent behavior

The Self-Report of Delinquency (SRD) assessed the number of times youth engaged in seven nonviolent and 10 violent crimes in the year prior to their incarceration [20]. Examples of nonviolent offenses included in the SRD scale are stealing a motor vehicle, stealing things worth more than $50, stealing things worth more between $5 and $50, stealing things worth less than $5, stealing marijuana or other drugs; examples of violent offenses included in the SRD scale are being in a gang fight, hitting a teacher, hitting a parent, threatening with force, forcing someone to have sex. For each item responses ranged from 0 (never) to 8 (two to three times a day). Summed scores could range from 0 to 136 (for this study *α* = 0.84).

#### Brief Symptom Inventory

The Brief Symptom Inventory (BSI), includes 53 items to evaluate the extent various thoughts or feelings “bothered or disturbed” youth (0 = not at all; 4 = extremely) “over the last seven days including today.” The BSI produces a Global Severity Index to assess overall current psychiatric distress (possible range = 0 to 212, for this study *α* = 0.96) and scores for the following symptom dimensions: somatization, obsessive-compulsive, interpersonal sensitivity, depression, anxiety, hostility, phobic anxiety, paranoid ideation, and psychoticism [21].

#### Psychopathic Personality Inventory-Short Version

The 56-item Psychopathic Personality Inventory-Short Version (PP1-SV) assessed to what extent personality characteristics described the youth (1 = false, 2 = mostly false, 3 = mostly true, 4 = true) [22]. The possible range was from 56 to 224 (for this study *α* = 0.76). In addition to the total score, the following subscales were included: Machiavellian Egocentricity, Carefree Nonplanfulness, Fearlessness, Blame Externalization, Impulsive Nonconformity, Stress Immunity.

#### Antisocial Process Screening Device

The Antisocial Process Screening Device (APSD, for this study *α* = 0.70) [23] is a scale consisting of 20-items that evaluate impulsivity, narcissism, and traits pertaining to being callous and unemotional.

#### Suicidal Ideation and attempts

The 5-item MAYSI- 2 Suicide Ideation scale [24] asks youth whether (yes = 1) they have (1) ever wished they were dead; (2) felt like life was not worth living, (3) felt like hurting themselves, (4) felt like killing themselves, and (5) ever given up hope for their life. This study’s α coefficient was .91. Additionally, youth were asked whether they “had ever actually tried to kill themselves” (yes = 1).

#### Substance use and related problems

Six measures assessed youths’ substance use and related problems. (1) Youth completed the MAYSI Alcohol/Drug Problems subscale [24]. (2) Youth provided the number of drug types they had used out of a list of 20 categories of psychoactive substances (e.g., cocaine). (3) They also indicated whether they had consumed alcohol, and (4) if they had consumed alcohol they indicated the age they began drinking alcohol. (5) Finally, they indicated whether they had consumed marijuana, and (4) if they had consumed marijuana they indicated the age they initiated marijuana use.

### Statistical Analyses

To examine patterns of inhalant use during one point in time this study carried out an exploratory latent class analysis (LCA) in Mplus 7.11. LCA combs through a dataset to identify and group together individuals with similar patterns of responses regarding types of inhalants used; the resulting groups are referred to as classes [25–29]. LCA’s underlying assumption is that the relationship among the dichotomous indicators can be explained by a categorical latent variable. Each dichotomous indicator is considered to be locally independent, which means that within each latent class the observed items are statistically independent [27, 30]. All models were estimated with 500 random starts to account for the fact that latent class models commonly have local maxima [31, 32]. Local maxima are peaks in the curve of the likelihood function prior to reaching the true global maximum, which is the actual highest point in the likelihood function [33].

Instead of testing an established or *a priori* class solution, seven models were evaluated based on model fit and heuristic utility. Overall model results and diagnostics were assessed for each latent class model using graphs of responses to questions screening types of inhalant use. Models were also chosen based on fit, interpretability and parsimony [34]. First, the one-class model was examined, and classes were increased until seven models had been evaluated. Information criteria compared the expected cell frequency count (*f*_*ijkl*_) and the observed cell frequency count (*F*_*ijkl*_) to assess model fit. Latent class models are considered unacceptable if observed data is too far from expected frequencies [33]. To determine the acceptability of each model the competing classes were evaluated using the most reliable information criteria, the sample-size adjusted Bayesian information criterion (SSA BIC) [32, 35, 36]. The likelihood-ratio statistic $G^{2}=2\sum_{w=1}^{W}f_{w}\text{log}(\frac{f_{w}}{{\hat{f}}_{w}})$, which takes the natural log of the observed values, *f*_w,_ and divides it by the expected values, ${\hat{f}}_{{}_{w}}$, is used to calculate the original unadjusted BIC (*BIC* = *G*2 + [log(*N*)|*p*), wherein log(N) is the natural log and P is the number of parameters in the model. BIC imposes a penalty on the *G* ^2^ so that as the sample size gets larger so does the penalty. As a result, the BIC’s ability to determine the correct model improves as the sample size gets larger [37]. For models with several parameters or small samples the SSA BIC replaces the *N* in the *BIC* formula, *BIC* = *G*2 + [log(*N*)|*p* with *N*’ = (*N* + 2)/24 to decrease the sample size penalty and improve performance [35, 37]. Rather than having a specific threshold, the lowest possible values are considered ideal for the SSA BIC [29]. We examined plots of SSA BIC for each class to determine which class had the best fit [26, 31, 32].

The entropy statistic is described by the formula $E=1-\frac{\Sigma_{i=1}^{n}\Sigma_{c=1}^{C}-p_{ic}\text{log}p_{ic}}{n\text{log}C}$, wherein *p*_*ic*_ is the posterior probability for individual *i*’s membership in class *c* and log denotes the natural log. Entropy gauged class purity or the extent that there was not error assigning individuals to a particular latent class [34]; ideally entropy values are as close to one as possible [26, 38].

Separate unadjusted analyses of 19 covariates using simple contrasts were conducted in Stata 13.1 using the following variables: race, geographic area of residence, gender, SRDProperty Crime Index, age at onset of offending, history of head injury, history of kidney disease, history of hormonal problems, history of mental illness, BSI-Global Severity Index, PPI-Fearlessness, PPI-Carefree Nonplanfulness, PPI-Impulsive Nonconformity, APSD-Callous/Unemotional Traits, APSDImpulsivity, MAYSI-2 Traumatic Experiences Scale, MAYSI-2 Suicide Ideation Scale,MAYSI-2 Alcohol/Drug Use Scales, and lifetime number of different drug types used.

## Results

### Sample Description

Sample characteristics are provided in Table 1, which compares inhalant users with non-users and provides results including effect sizes for differences. The full sample was 87% male, 56% White, and 39% urban. Given that only 13% of the sample was female, these results may more accurately explain male patterns of inhalant use compared to female patterns. That said, it is noteworthy that females were disproportionately represented among inhalant users.

**Table 1:** Sample Characteristics of 723 Incarcerated Youth.

	Inhalant Users			Non-Users			Full Sample			Results Comparing Inhalant Users and Non-Users
	*N*	*M*/%	S.D.	*N*	*M*/%	S.D.	*N*	*M*/%	S.D.	
Age	287	15.5	1.1	436	15.5	1.3	723	15.5	1.2	F (1, 664.9) ^a^ = 0.9, p = 0.334^b^

										η ^c^ = 0.00, CI [., 0.01] ^d^
Sex										
Male	241	84.0		388	89.0		629	87.0		χ2 (1) = 3.9;
Female	46	16.0		48	11.0		94	13.0		p = 0.050;
										Cramer's V^e^ = 0.07, CI[., 0.2]
**Race/Ethnicity**										
**White**	**216**	**75.3**		**184**	**42.2**		**400**	**55.4**		**χ2 (4)** ^f^ **= 156.6;**
**Black**	**23**	**8.0**		**215**	**49.3**		**238**	**33.0**		**p = 0.000**
**Hispanic**	**20**	**7.0**		**8**	**1.8**		**28**	**3.9**		**Cramer's V = 0.44,**
**Biracial**	**20**	**7.0**		**25**	**5.7**		**45**	**6.2**		**CI[0.37, 0.51]**
**Other**	**7**	**2.4**		**4**	**0.9**		**11**	**1.5**		
**Missing**	**1**	**0.4**		**0**	**0**		**1**	**0.0**		
**Geographic Area**										
**Urban City**	**78**	**27.2**		**205**	**47.0**		**283**	**39.1**		**χ2 (3) = 33.9; p = 0.000**
**Suburban Area**	**41**	**14.3**		**59**	**13.5**		**100**	**13.8**		**Cramer's V = 0.21,**
**Small Town**	**147**	**51.2**		**139**	**31.9**		**286**	**39.6**		**CI[0.15, 0.29]**
**Rural/Country**	**21**	**7.3**		**33**	**7.6**		**54**	**7.5**		
Receives Welfare										χ2 (1) = 0.3;
Yes	111	38.7		177	40.6		288	39.8		p = 0.580
No	173	60.3		253	58.0		426	58.9		Cramer's V = -0.02,
Missing	3	1.1		6	1.4		9	1.2		CI[., 0.10]
Physical and mental health N (%)										
Head injury caused blackout										χ2 (1) = 6.9; p = 0.008
Yes	221	77.0		66	15.1		132	18.3		Cramer's V = 0.10,
No	66	23.0		367	84.2		588	81.3		CI[0.04, 0.18]
Missing	0	0		3	0.7		3	0.4		
**Diagnosed with mental illness**										
**Yes**	**186**	**64.8**		**184**	**42.2**		**370**	**51.2**		**χ2 (1) = 35.4; p = 0.000**
**No**	**100**	**34.8**		**250**	**57.3**		**350**	**48.4**		**Cramer's V = 0.22,**
**Missing**	**1**	**0.4**		**2**	**0.5**		**0**	**0.41**		**CI[0.15, 0.30]**
**Heard voices of people not there**										
**Yes**	**59**	**20.6**		**47**	**10.8**		**106**	**14.7**		**χ2 (1) = 13.2; p = 0.000**
**No**	**228**	**79.4**		**389**	**89.2**		**617**	**85.3**		**Cramer's V = 0.14,**
										**CI[0.07, 0.21]**
Delinquent behavior M (S.D.)										
**Self-Report of**	**287**	**27.5**	**18.9**	**436**	**22.4**	**17.9**	**723**	**24.4**	**18.5**	**F (1,721) = 13.6, p = 0.0002**
**Delinquency (SRD)**										**η = 0.02, CI [0.00, 0.04]**
**Total delinquency**										
SRD Property	287	16.7	12.0	436	12.3	11.4	723	14.0	11.8	**F (1,721) = 25.0, p = 0.0000**
										**η = 0.03, CI [0.01, 0.06]**
Age at onset of	286	10.1	2.9	435	10.7	2.9	721	10.5	2.9	F (1,719) = 7.2, p = 0.0073
offending (years)										η = 0.01, CI [0.00, 0.03**]**
**Brief Symptom Inventory (BSI) M (S.D.)**										
**Global Severity**	**282**	**53.9**	**36.7**	**425**	**37.0**	**31.8**	**707**	**43.7**	**34.8**	**F (1,541.1) = 39.8, p = 0.000**
**Index**										**η = 0.06, CI [0.03, 0.09]**
**Somatization**	**287**	**4.4**	**4.6**	**436**	**3.1**	**4.1**	**723**	**3.6**	**4.4**	**F (1,556.1) = 15.3, p = 0.000**
										**η = 0.02, CI [0.01, 0.05]**
**Obsessive–**	**287**	**8.3**	**5.7**	**436**	**5.5**	**4.9**	**723**	**6.6**	**5.4**	**F (1,545.3) = 48.7, p = 0.000**
**Compulsive**										**η = 0.07, CI [0.04, 0.10]**
**Interpersonal**	**287**	**3.5**	**3.7**	**436**	**2.4**	**3.1**	**723**	**2.9**	**3.4**	**F (1,556.1) = 15.3, p =**
**Sensitivity**										**0.0001**
										**η = 0.02, CI [0.01, 0.05]**
**Depression**	**287**	**6.1**	**5.5**	**436**	**3.8**	**4.5**	**723**	**4.7**	**5.0**	**F (1,521.8) = 35.5, p = 0.000**
										**η = 0.05, CI [0.02, 0.09]**
**Anxiety**	**287**	**5.7**	**5.2**	**436**	**3.5**	**4.2**	**723**	**4.4**	**4.7**	**F (1,519.9) = 37.1, p = 0.000**
										**η = 0.05, CI [0.03, 0.09]**
**Hostility**	**287**	**6.9**	**5.1**	**287**	**6.9**	**5.1**	**723**	**6.1**	**5.0**	**F (1,721) = 15.5, p = 0.0001**
										**η = 0.02, CI [0.01, 0.05]**
**Phobic Anxiety**	**287**	**2.5**	**3.4**	**436**	**1.7**	**3.1**	**723**	**2**	**3.2**	**F (1,571.2) = 9.3, p = 0.0024**
										**η = 0.01, CI [0.00, 0.03]**
**Paranoid Ideation**	**287**	**7.2**	**4.7**	**436**	**5.7**	**4.6**	**723**	**6.3**	**4.7**	**F (1, 721) = 19.6, p = 0.000**
										**η = 0.03, CI [0.01, 0.05]**
**Psychoticism**	**287**	**4.7**	**4.4**	**436**	**2.9**	**3.3**	**723**	**3.6**	**3.9**	**F (1, 489.9) = 32.8, p = 0.000**
										**η = 0.05, CI [0.02, 0.08]**
**Psychopathic Personality Inventory M (S.D.)**										
**Total**	**287**	**141.5**	**14.9**	**436**	**133.1**	**12.3**	**723**	**136.4**	**14.0**	**F (1,530.2) = 62.9, p = 0.000**
										**η = 0.07, CI [0.03, 0.10]**
Machiavellian	287	17.9	4.4	436	16.8	4.4	723	17.2	4.5	F (1,721) = 10.2, p = 0.0014
Egocentricity										η = 0.01, CI [0.00, 0.04]
**Carefree**	**287**	**15.4**	**3.8**	**436**	**13.5**	**3.8**	**723**	**14.2**	**3.9**	**F (1, 721) = 44.3, p = 0.000**
**Nonplanfulness**										**η = 0.06, CI [0.03, 0.09]**
**Fearlessness**	**287**	**19.3**	**4.8**	**436**	**15.6**	**5.0**	**723**	**17.1**	**5.3**	**F (1, 721) = 96.6, p = 0.000**
										**η = 0.12, CI [0.08, 0.16]**
Blame Externalization	287	18.4	4.6	436	17.8	4.9	723	18.2	4.8	F (1, 643.2) = 7.9, p = 0.0059
										η = 0.01, CI [0.00, 0.03]
**Impulsive**	**287**	**16.0**	**4.5**	**436**	**14.0**	**3.6**	**723**	**14.8**	**4.1**	**F (1,514.9) = 40.5, p = 0.000**
**Nonconformity**										**η = 0.06, CI [0.03, 0.09]**
**Stress Immunity**	**287**	**18.4**	**4.4**	**436**	**19.4**	**4.3**	**723**	**19.0**	**4.3**	**F (3, 719) = 7.3, p = 0.0001**
										**η = 0.03, CI [0.01, 0.05]**
**Antisocial Process Screening Device M (S.D.)**										
**Total**	**287**	**17.7**	**5.3**	**435**	**15.3**	**5.4**	**722**	**16.3**	**5.5**	**F (1, 720) = 32.0, p = 0.000**
										**η = 0.04, CI [0.02, 0.07]**
**Callous/Unemotional**	**287**	**8.2**	**3.2**	**435**	**7.3**	**3.0**	**722**	**7.7**	**3.1**	**F (1, 720) = 15.8, p = 0.0001**
**Traits**										**η = 0.02, CI [0.01, 0.05]**
**Impulsivity**	**287**	**7.2**	**1.9**	**435**	**6.0**	**2.2**	**722**	**6.5**	**2.2**	**F (1, 720) = 54.3, p = 0.000**
										**η = 0.07, CI [0.04, 0.11]**
**Massachusetts Youth Screening Inventory M (S.D.)**										
**Traumatic**	**287**	**3.2**	**1.6**	**435**	**2.8**	**1.6**	**722**	**3.0**	**1.6**	**F (1, 720) = 12.1, p = 0.0005**
**Experiences**										**η = 0.02, CI [0.00, 0.04]**
**Suicidal Ideation** ^**a**^	**287**	**3.2**	**2.4**	**435**	**1.6**	**2.1**	**722**	**2.2**	**2.4**	**F (1, 543.7) = 85.3, p = 0.000**
										**η = 0.11, CI [0.07, 0.16]**
**Substance use and related problems**										
**MAYSI-**	**287**	**5.2**	**2.1**	**423**	**3.2**	**5.2**	**710**	**4.0**	**4.4**	**F (1, 708) = 36.4, p = 0.000**
**Alcohol/Drug**										**η = 0.05, CI [0.02, 0.08]**
**Problems M (S.D.)**										
**Lifetime # of drug**	**287**	**5.9**	**2.9**	**434**	**2.8**	**2.0**	**721**	**4.0**	**2.8**	**F (1, 719) = 294.9, p = 0.000**
**types used M (S.D.)**										**η = 0.29, CI [0.24, 0.34]**
**Lifetime alcohol use**	**274**	**95.5**		**339**	**77.8**		**613**	**84.8**	**2.7**	**χ2 (1) = 42.1, p = 0.000**
**N (%)**										**Cramer’s V = 0.24, CI [0.17,**
										**0.32]**
**Age at onset of**	274	11.2	2.8	339	11.9	2.6	613	11.6	2.7	F (1, 611) = 9.5, p = 0.0022
**alcohol use (years)**										η = 0.02, CI [0.00, 0.04]
**M (S.D.)**										
**Lifetime marijuana**	**274**	**95.5**		**352**	**80.7**		**626**	**86.6**		**χ2 (1) = 32.4, p = 0.000**
**use N (%)**										**Cramer’s V = 0.21, CI**
										**[0.14, 0.29]**
Age at onset of	274	11.0	2.4	351	11.5	2.1	625	11.3	2.2	F (1, 623) = 6.7, p = 0.0101
marijuana use (years)										η = 0.01, CI [0.00, 0.03]
M (S.D.)										

Note. M = mean; S.D. = standard deviation.

^a^ In cases where violations of homogeneity of variance assumptions necessitated more stringent tests for statistical significance, statistical contrasts associated with fractional degrees of freedom are provided (c.f., http://www.ats.ucla.edu/stat/stata/library/homvar.htm).

^b^ Due to the number of tests conducted, a Bonferroni alpha correction was calculated using the multproc add-on in Stata [39]; the resulting alpha is 0.0013. Bolded text indicates significance after applying the alpha correction.

^c^ η = Eta-squared effect size for more than two independent groups was computed using ANOVA values and associated degrees of freedom (c.f., http://blog.stata.com/2013/09/05/measures-of-effect-size-in-stata-13/).

^d^ Because eta-squared ranges from 0 to 1 [40] confidence intervals cannot include values outside these bounds, a value outside these bounds was entered as a missing value.

^e^ Cramer’s V is an effect size that measures association between two nominal variables. It ranges from 0 to 1; 1 indicates strong association.

^f^ When cell sizes were below 5 in more than 20% of the cells, the likelihood-ratio chi-square test was used.

Table 2 provides the prevalence of using each inhalant. For both the full sample and the inhalant user subsample the most frequently used inhalant was gasoline (22% and 57% respectively), and the least frequently used inhalant was using airplane or model glue (5% and 14% respectively).

**Table 2:** Lifetime Prevalence of Inhalant Use Behaviors of 723 Incarcerated Youth.

	Full Sample		Inhalant Users	
	*N*	%	*N*	%
Airplane or model glue	39	5.4	39	14.0
Anesthetic gases	45	6.2	45	16.1
Freon ^a^	44	6.1	44	15.8
Gas from whipping cream cans	44	6.1	44	15.8
Butane ^b^	50	6.9	50	17.9
"White-out" or another correction fluid	52	7.2	52	18.6
Air Freshener ^c^	58	8.0	58	20.8
Nail polish	61	8.4	61	21.9
Nail polish remover	63	8.7	63	22.6
Whippets ^d^	65	9.0	65	23.3
Spray paint	83	11.5	83	29.8
Gases from computer "duster" sprays	106	14.7	106	38.0
Permanent markers	106	14.7	106	38.0
Gasoline	159	22.0	159	57.0

^a^ Freon can come from an air conditioner or other appliance.

^b^ Examples of butane include cigarette or cigar lighter gas.

^c^ An example of an air freshener is “Glade.”

^d^ Whippets are carbon dioxide (CO2) canisters that contain Nitrous Oxide.

Table 3 provides the results of the LCA for each of the seven classes. The top row provides the fit and heuristic indices used to evaluate the models, as well as the distribution of individuals in each class presented as numbers and percentages. The number of classes are indicated on the left-hand side of the table. The fit statistics indicated that the four-class model had the best fit. The four-class solution was chosen based on the best fit, interpretability and its parsimonious fit of the data. The distribution of the residuals for the four-class model did not indicate that the local independence assumption was violated.

**Table 3:** Indicators of Fit for One through Seven Classes (N = 723).

Full Sample				
	BIC SSA	Entropy	Class n	Class %
1	6258.8		723	100.0%
2	5003.8	0.9	206	28.5%
			517	71.5%
3	4934.8	0.9	83	11.5%
			124	17.2%
			516	71.4%
**4**	**4892.2**	**0.9**	**50**	**6.9%**
			**77**	**10.7%**
			**83**	**11.5%**
			**513**	**71.0%**
5	4901.8	0.9	21	2.9%
			30	4.1%
			76	10.5%
			83	11.5%
			513	71.0%
6	4916.1	0.9	4	0.6%
			14	1.9%
			40	5.5%
			73	10.1%
			79	10.9%
			513	71.0%
7	4941.5	0.9	4	0.6%
			11	1.5%
			15	2.1%
			27	3.7%
			74	10.2%
			88	12.2%
			504	69.7%

Note. BIC SSA = sample-size-adjusted Bayesian Information Criteria. The SSA BIC places a lower penalty on added parameters based on sample size than the BIC, and is useful for smaller sample sizes. The four-class model is bold-faced to indicate that it was the model chosen for the full sample.

**Fig 1:**
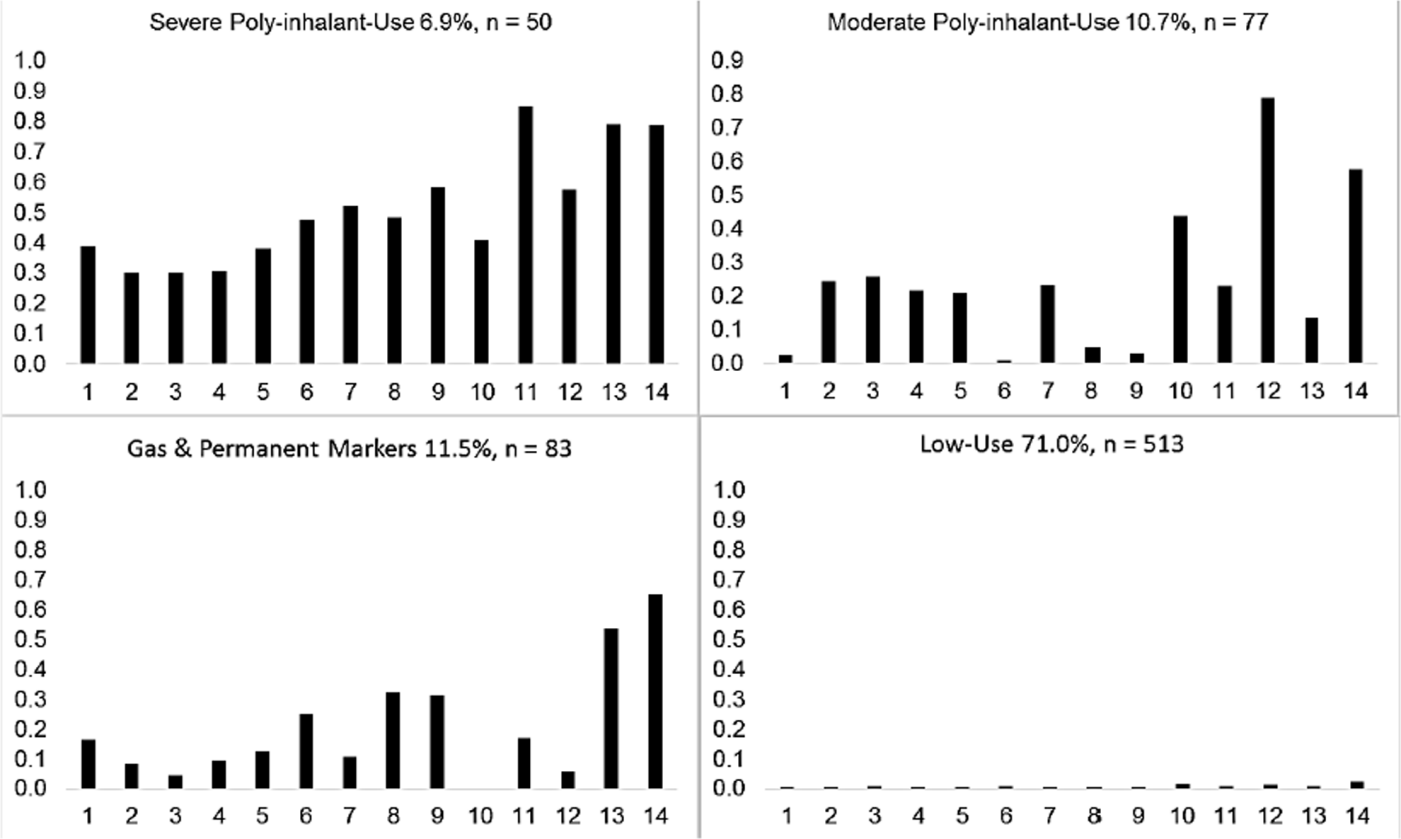
Plot of Polyinhalant Use Classes. Note. (1) Airplane or model glue; (2) Anesthetic gases; (3) Freon; (4) Gas from whipping cream cans; (5) Butane; (6) "White-out" or another correction fluid; (7) Air Freshener; (8) Nail polish; (9) Nail polish remover; (10) Whippets; (11) Spray paint; (12) Gases from computer "duster" sprays; (13) Permanent markers; (14) Gasoline.

Fig 1 depicts the item-response probabilities for using each of the 14 inhalants among the full sample. The four classes reflect (1) severe polyinhalant use; (2) moderate polyinhalant use; (3) gas and permanent marker use; and (4) low-use.

Results of the univariate contrasts are provided in Table 4. Members of the four classes did not differ with regard to gender or welfare receipt. Greater proportions of youth who were White were in the severe polyinhalant use class compared to the low-use class (80% and 47% respectively). Most youth not in the low-use class lived outside of urban areas. Differences were not found between the classes with regard to family welfare receipt. Post-hoc analyses were conducted to examine bivariate differences; those results are provided in Tables A and B in S1 File.

**Table 4:** Full Sample unadjusted univariate contrasts of severe poly-inhalant users (N = 50), moderate poly-inhalant users (N = 77), gas and permanent marker users (N = 83), and low-user class members (N = 513) across criminological, health, mental health, attitudinal, and substance use measures.

Variables	Severe Poly-inhalant Use	Moderate Poly-inhalant Use	Gas & Perm. Markers Use	Low-Use	Results
Age M (S.D.)	15.7 (0.8)	15.8 (0.9)	15.2 (1.4)	15.5 (1.3)	F (3, 234.5) ^a^ = 4.6, p = 0.0039 ^b^
					η ^c^ = 0.02, CI [0.00, .03]
Sex N (%)					
Male	41 (82.0)	67 (87.0)	70 (84.3)	451 (87.9)	χ2 (3) = 2.0, p = 0.571; Cramer's V ^d^ = 0.05,
Female	9 (18.0)	10 (13.0)	13 (15.7)	62 (12.1)	CI[., 0.12]
**Race/Ethnicity N (%)**					
**White**	**39 (78.0)**	**62 (80.5)**	**58 (69.9)**	**241 (47.0)**	**χ2 (3)** ^**e**^ **= 133.5, p = 0.000;**
**Black**	**2 (4.0)**	**2 (2.6)**	**9 (10.8)**	**225 (43.9)**	**Cramer's V = 0.23,**
**Hispanic**	**4 (8.0)**	**6 (7.8)**	**4 (4.8)**	**14 (2.7)**	**CI[0.19, 0.28]**
**Biracial**	**3 (6.0)**	**7 (9.1)**	**6 (7.2)**	**29 (5.7)**	
**Other**	**1 (2.0)**	**0 (0)**	**6 (7.2)**	**4 (0.8)**	
**Missing**	**1 (2.0)**	**0 (0)**	**0 (0)**	**0 (0)**	
**Geographic Area N (%)**					
**Urban City**	**18 (36.0)**	**18 (23.4)**	**18 (21.7)**	**229 (44.6)**	**χ2 (3) = 34.6, p = 0.000;**
**Suburban Area**	**8 (16.0)**	**8 (10.4)**	**11 (13.3)**	**73 (14.2)**	**Cramer's V = 0.13,**
**Small Town**	**20 (40.0)**	**46 (59.7)**	**45 (54.2)**	**175 (34.1)**	**CI[0.09, 0.17]**
**Rural/Country**	**4 (8.0)**	**5 (6.5)**	**9 (10.8)**	**36 (7.0)**	
Receives Welfare N (%)					
Yes	18 (36.0)	29 (37.7)	37 (45.1)	204 (40.4)	χ2 (3) = 1.4, p = 0.706; Cramer's V = 0.04,
Missing	0 (0)	0 (0)	1 (1.2)	8 (1.56)	CI[0.09, 0.17]
Physical and mental health N (%)					
History of head injury					
Yes	17 (34.0)	18 (23.4)	18 (21.7)	79 (15.5)	χ2 (3) = 12.9, p = 0.005; Cramer's V = 0.13,
Missing	0 (0)	0 (0)	0 (0)	3 (0.6)	CI[., 0.12]
**Mental Illness Diagnosis**					
**Yes**	**42 (85.7)**	**51 (66.2)**	**55 (66.3)**	**222 (43.4)**	**χ2 (3) = 50.2, p = 0.000; Cramer's V = 0.26,**
**Missing**	**1 (2.0)**	**0 (0)**	**0 (0)**	**2 (0.4)**	**CI[0.20, 0.34]**
**Hearing voices**					
**Yes**	**15 (30.0)**	**11 (14.3)**	**25 (30.1)**	**55 (10.7)**	**χ2 (3) = 31.6, p = 0.000; Cramer's V = 0.21,**
					**CI[0.20, 0.34]**
Delinquent behavior M (S.D.)					
**SRD (Total**	**36.2(18.9)**	**26.2(19.4)**	**26.4(18.2)**	**22.7(17.9)**	**F (3, 231.7) = 8.6, p = 0.000;**
**Delinquency)**					**η = .04, CI [0.01, .06]**
**SRD (Property**	**21.8(11.4)**	**16.0(12.3)**	**15.7(10.9)**	**12.7(11.6)**	**F (3, 719) = 11.0, p = 0.000; η = .04,**
					**CI [0.02, 0.07]**
Age at onset of	9.5 (2.8)	10.1 (3.0)	10.1 (2.9)	10.7 (2.9)	F (3, 717) = 4.1, p = 0.0064; η = .02,]
offending (years)					CI [0.02, 0.07
Brief Symptom Inventory M (S.D.)					
**Global Severity**	**79.9(41.4)**	**47.8 (32.7)**	**55.2(38.4)**	**37.6 (30.9)**	**F (3, 200.0) = 23.2, p = 0.000; η = .11**
**Index**					**CI [0.07, 0.15]**
**Somatization**	**7.5 (6.0)**	**3.4 (3.7)**	**4.8 (4.7)**	**3.1 (4.0)**	**F (3, 165.1) = 14.9, p = 0.000; η = .08,**
					**CI [0.04, 0.11]**
**Obsessive–**	**12.5 (6.1)**	**8.0 (5.9)**	**8.0 (5.2)**	**5.6 (4.8)**	**F (3, 208.1) = 34.2, p = 0.000; η = .12,**
**Compulsive**					**CI [0.08, 0.17]**
**Interpersonal**	**5.3 (4.4)**	**2.6 (2.9)**	**4.1 (4.1)**	**2.5 (3.1)**	**F (3, 186.8) = 15.0, p = 0.000; η = .06,**
**Sensitivity**					**CI [0.03, 0.09]**
**Depression**	**9.6 (6.5)**	**5.7 (5.6)**	**6.0 (5.5)**	**3.9 (4.4)**	**F (3, 195.9) = 17.5, p = 0.000; η = .09,**
					**CI [0.06, 0.13]**
**Anxiety**	**9.4 (5.8)**	**5.2 (5.0)**	**5.5 (5.2)**	**3.6 (4.1)**	**F (3, 202.3) = 21.0, p = 0.000; η = .11,**
					**CI [0.07, 0.15]**
**Hostility**	**8.9 (5.4)**	**6.7 (4.9)**	**7.1 (5.0)**	**5.5 (4.8)**	**F (3, 219.3) = 8.7, p = 0.000; η = .04,**
					**CI [0.01, 0.07]**
**Phobic Anxiety**	**4.2 (4.5)**	**1.8 (2.6)**	**2.6 (3.4)**	**1.7 (3.0)**	**F (3, 158.1) = 8.5, p = 0.000; η = .04,**
					**CI [0.02, 0.07]**
**Paranoid Ideation**	**9.2 (4.6)**	**6.7 (4.9)**	**7.7 (5.2)**	**5.7 (4.5)**	**F (3, 238.9) = 11.3, p = 0.000; η = .05,**
					**CI [0.02, 0.08]**
**Psychoticism**	**7.3 (4.7)**	**4.3 (4.2)**	**4.7 (4.3)**	**3.0 (3.4)**	**F (3, 207.8) = 17.9, p = 0.000; η = .09,**
					**CI [0.05, 0.13]**
Psychopathic Personality Inventory M (S.D.)					
**Total**	**143.2 (13.9)**	**142.2 (15.9)**	**141.1(13.7)**	**134.1(13.2)**	**F (3, 719) = 16.8 p = 0.000 η = .07,**
					**CI [0.03, 0.10]**
**Machiavellian**	**19.5 (4.1)**	**16.7 (4.2)**	**18.4 (4.7)**	**16.9 (4.4)**	**F (3, 719) = 7.4 p = 0.000; η = .03,**
**Egocentricity**					**CI [0.01, 0.06]**
**Carefree**	**16.3 (4.4)**	**15.3 (3.8)**	**15.4 (3.8)**	**13.7 (3.8)**	**F (3, 719) = 13.3, p = 0.000; η = .05,**
**Nonplanfulness**					**CI [0.02, 0.08]**
**Fearlessness**	**19.9 (4.8)**	**19.4 (5.2)**	**19.9 (4.6)**	**16.0 (5.0)**	**F (3, 719) = 27.7, p = 0.000; η = .10,**
					**CI [0.06, 0.14]**
Blame Externalization	20.2 (4.1)	18.4 (4.8)	19.2 (4.7)	17.9 (4.8)	F (3, 719) = 5.2, p = 0.015; η = .02,
					CI [0.00, 0.04]
**Impulsive**	**17.3 (4.5)**	**15.6 (4.4)**	**16.3 (4.2)**	**14.2 (3.8)**	**F (3, 719) = 15.4, p = 0.000; η = .06,**
**Nonconformity**					**CI [0.03, 0.14]**
**Stress Immunity**	**16.9 (3.9)**	**19.2 (4.6)**	**17.9 (4.4)**	**19.3 (4.3)**	**F (3, 719) = 7.3, p = 0.0001; η = .03,**
					**CI [0.01, 0.05]**
**Antisocial Process Screening Device M (S.D.)**					
**Total**	**18.9 (5.2)**	**17.2 (5.0)**	**18.3 (5.8)**	**15.6 (5.4)**	**F (3, 718) = 11.4, p = 0.000; η = .05,**
					**CI [0.02, 0.08]**
Callous/Unemotional Traits	8.3 (3.5)	8.0 (3.0)	8.7 (3.4)	7.4 (3.0)	F (3, 718) = 5.3, p = 0.003; η = .02,
					CI [0.00, 0.04]
**Impulsivity**	**8.1 (1.7)**	**7.4 (2.0)**	**7.1 (2.0)**	**6.0 (2.1)**	**F (3, 718) = 23.5, p = 0.000; η = .09,**
					**CI [0.05, 0.13]**
Massachusetts Youth Screening Inventory M (S.D.)					
**Traumatic**	**4.1 (1.1)**	**3.0 (1.6)**	**3.3 (1.5)**	**2.8 (1.6)**	**F (3, 264.7) = 14.9, p = 0.000; η = .05,**
**Experiences**					**CI [0.02, 0.08]**
**MAYSI-Suicide**	**4.5 (2.1)**	**2.6 (2.3)**	**3.5 (2.4)**	**1.7 (2.2)**	**F (3, 244.4) = 35.7, p = 0.000; η = .14,**
**Ideation**					**CI [0.09, 0.18]**
Substance use and related problems					
**MAYSI-**	**6.4 (1.7)**	**5.5 (1.7)**	**4.9 (2.4)**	**3.4 (4.9)**	**F (3, 706) = 13.0, p = 0.000; η = .05,**
**Alcohol/Drug**					**CI [0.02, 0.08]**
**Problems M (S.D.)**					
**Lifetime # of drug**	**7.2 (3.0)**	**7.4 (2.3)**	**4.7 (2.7)**	**3.1 (2.2)**	**F (3, 198.3) = 92.3, p = 0.000; η = .32,**
**types used M (S.D.)**					**CI [0.27, 0.37]**
**Lifetime alcohol use**	**49 (98.0)**	**75 (97.4)**	**78 (94.0)**	**411 (80.2)**	**χ2 (3) = 38.7, p = 0.000; Cramer's V = 0.21,**
**N (%)**					**CI [0.14, 0.27]**
**Age at onset of alcohol**	**10.8 (2.8)**	**11.0 (2.9)**	**10.8 (3.0)**	**12.0 (2.5)**	**F (3, 230.2) = 7.3, p = 0.000; η = .04;**
**use (years) M (S.D.)**					**CI [0.01, 0.07]**
**Lifetime marijuana**	**47 (94.0)**	**77(100.0)**	**76 (91.6)**	**426 (83.0)**	**χ2 (3) = 32.3, p = 0.000; Cramer's V = 0.17,**
**use N (%)**					**CI[0.11, 0.25]**
**Age at onset of marijuana use (years) M (S.D.)**					
	**10.7 (2.3)**	**10.3 (2.5)**	**11.4 (2.2)**	**12.0 (2.5)**	**F (3) = 8.1, p = 0.000; η = .04, CI[0.01, 0.07]**

Note. M = mean; S.D. = standard deviation.

^a^ In cases where violations of homogeneity of variance assumptions necessitated more stringent tests for statistical significance, statistical contrasts associated with fractional degrees of freedom are provided (c.f., http://www.ats.ucla.edu/stat/stata/library/homvar.htm).

^b^ Due to the number of tests conducted, a Bonferroni alpha correction was calculated using the multproc add-on in Stata [39]; the resulting alpha is 0.0013. Bolded text indicates significance after applying the alpha correction.

^c^ η = The eta-squared effect size for more than two independent groups, which ranges from 0 to 1 [40], was computed using ANOVA values and associated degrees of freedom (c.f., http://blog.stata.com/2013/09/05/measures-of-effect-size-in-stata-13/). When values fall outside the bounds of 0 and 1 they are represented as a missing value.

^d^ Cramer’s V is an effect size that measures association between two nominal variables. It ranges from 0 to 1; 1 indicates strong association [41]. When values fall outside the bounds of 0 and 1 they are represented as a missing value.

^e^ When cell sizes were below 5 in more than 20% of the cells the likelihood-ratio chi-square test was used.

Compared to the low-use class, members of the severe polyinhalant use class had experienced more than double the rate of TBI (34.0% vs. 15.5%). Youth in the severe polyinhalant class had the highest rates of mental illness diagnoses. Furthermore, mental illness diagnoses accounted for 26% of the variance. The gas and markers class had the highest rate of hearing voices, followed by the severe polyinhalant use class, and the moderate polyinhalant use class.

Delinquent behavior was also higher in the severe polyinhalant use class and began younger than in the other classes. Scores on the BSI indicated more severe impairment for youth in the severe polyinhalant use class compared to the other classes. Members of the gas and markers class had the second highest scores, followed by the moderate polyinhalant class. With the exception of the obsessive compulsive subscale, members of the severe polyinhalant class had the highest scores on the BSI subscales followed by members of the gas and marker class, then by members of the moderate polyinhalant class. Class members in the severe polyinhalant class had the highest scores on the Psychopathic Personality Inventory, followed by members of the gas and markers class, and the moderate polyinhalant class. Compared to low-use class members, youth in the inhalant use classes scored higher on the Antisocial Process Screening Device and its subscales. Traumatic experiences and suicidal ideation were notably higher for members of the three inhalant use classes compared to the low-use class. Members of the three Inhalant use classes had more substance use related problems, with the greatest problems being among the severe polyinhalant use class. Members of the severe polyinhalant and gas and marker classes initiated alcohol use at the same time, while members of the moderate polyinhalant class had the earliest onset of marijuana use.

## Discussion

The resulting four-class model consisted of (1) severe polyinhalant use; (2) moderate polyinhalant use; (3) gas and permanent marker use; and (4) low-use. The classes are ordered from smallest to largest, with the smallest class being the severe polyinhalant users and the largest class being low-users. Group differences did not exist based on age, sex, or receipt of welfare. However, given the small sample of females (N = 94, 13%) the findings pertaining to sex are necessarily tentative. Members of the severe polyinhalant class were more likely to be White and to live outside of urban areas compared to members of the low-use class. It may be that youth in metropolitan areas have greater exposure to inhalants. This access may be the result of a somewhat higher socioeconomic status. For example, it seems that homes with a computer would be more likely to have computer duster sprays than those without a computer. Likewise, residents in a home with a lawnmower may be more far likely to have gasoline than residents in an apartment in an urban area.

In unadjusted analyses, the three classes of inhalant users had higher rates of self-reported physical and mental health problems, higher rates of delinquency, an earlier age of initiating delinquency, worse psychological impairment, significantly higher scores on each of the subscales of the Psychopathic Personality Inventory, higher total scores on the Antisocial Process Screening Device and each of its subscales, scored worse on the traumatic experiences and suicidal ideation subscales of the MAYSI instrument, and reported more substance use and related problems compared to the low-using class.

Consistent with other studies that have found higher rates of TBI among inhalant users [2], when we used a conventional threshold (p = 0.05) we found that youth in the three inhalant use classes experienced higher rates of TBI than youth in the low-use class. Of the four classes, the severe polyinhalant use class had the highest proportion of youth with head injuries. These findings add further support to literature, which indicates that neurocognitive impairments contribute to substance use and other risk behaviors [12, 17]. It seems likely that effects of TBI, including slower perceptual and motor processing, lower IQ and heightened impulsivity [14], contribute to this outcome. It is important to note that after the alpha correction was applied these results were no longer significant. We believe that the differences in proportions merit further investigation. Future studies should seek to further elucidate the relationship between TBI and inhalant use.

Our findings suggest that youth in the severe polyinhalant class are especially afflicted by high rates of psychiatric disorders. These findings are similar to those found in both population-based studies [7] and studies of incarcerated youth [2]. Compared to youth in the low-use class, we found that a greater proportion of youth in the severe polyinhalant use class had been diagnosed with a mental illness, and had reported hearing voices of people who were not there. Interestingly, members of the gas and markers class are more psychiatrically disordered than the moderate polyinhalant user group. The risk of suicidality among inhalant users is particularly disconcerting, and echoes findings of prior studies [2,11]. The linkage between mental health impairment and inhalant use raises the question of whether youth using inhalants may be self-medicating to address an underlying condition.

In addition, similar to prior studies using both population-based data and incarcerated youth, we found that youth in the inhalant use classes had used more substances and experienced more problems with their substance use than youth in the low-use class. This finding suggests that inhalant users may be particularly vulnerable to the adverse consequences of substance use, including experiencing drug interactions or engaging in high-risk behaviors.

This study has important implications for policy and practice. Early prevention strategies are needed to reduce the risk of polyinhalant use among youth who have experienced a head injury. Additionally, both policymakers and clinicians should target antisocial youth for prevention and treatment. In particular, relatively low-cost treatment interventions could be implemented within adolescent correctional facilities, juvenile detention centers, and adolescent substance abuse treatment service providers. Clinicians treating youth with severe psychiatric conditions should assess for inhalant use.

## Conclusions

This study is the first to investigate how the use of different inhalants co-occur, and how these different classes of youth vary with regard to medical conditions, psychiatric symptoms, personality traits and substance use behaviors. Study strengths included the high participation rate; the inhalant use assessment with several different psychoactive inhalants; and the methodology, which shed light on patterns of polyinhalant use among incarcerated youth. Study limitations include the cross-sectional nature of the data, relying on self-report measures without validating data through other means, and the potential limited generalizability of using a criminological sample. Additionally, because the VSSI was first used in this study, no reliability and validity data are available for this measure. That said, many of the study results were consistent with findings obtained in general population surveys.

Both policymakers and clinicians should target antisocial youth for prevention and treatment. Future studies should explore how inhalant use changes over time, and clarify whether inhalant use is a form of self-medication, or if inhalant use contributes to mental health conditions, such as anxiety, depression, psychosis, or obsessive-compulsive disorder. Qualitative studies could help further our understanding of the reasons antisocial youth are especially vulnerable to inhalant use.

## Supporting Information

S1 File (DOCX)
