# Supplementary material for: Patterns of Inhalant Use among Incarcerated Youth
Source: PLoS One. 2015 Sep 2;10(9):e0135303. doi: 10.1371/journal.pone.0135303 (PMC4557982; doi:10.1371/journal.pone.0135303)
Supplement: S1 File — (DOCX) [file pone.0135303.s001.docx]

Supplemental Tables

**Table A. Bonferroni post-hoc comparisons of bivariate relationships with continuous variables**

| **Variable** |  | **Class Comparisons** |  | **Mean Diff.** |  | **S.E.** |  | **t** |  | **p-value** |
| --- | --- | --- | --- | --- | --- | --- | --- | --- | --- | --- |
| Age |  |  |  |  |  |  |  |  |  |  |
|  |  | Moderate Poly-inhalant Use vs. Severe Poly-inhalant Use |  | 0.1 |  | 0.2 |  | 0.6 |  | 1.000 |
|  |  | Gas & Perm. Markers Use vs. Severe Poly-inhalant Use |  | -0.5 |  | 0.2 |  | -2.8 |  | 0.122 |
|  |  | Low-Use vs. Severe Poly-inhalant Use |  | -0.2 |  | 0.1 |  | -1.4 |  | 1.000 |
|  |  | **Gas & Perm. Markers Use vs. Moderate Poly-inhalant Use** |  | **-0.6** |  | **0.2** |  | **-3.6** |  | **0.010** |
|  |  | Low-Use vs. Moderate Poly-inhalant Use |  | -0.3 |  | 0.1 |  | -2.5 |  | 0.304 |
|  |  | Low-Use vs. Gas & Perm. Markers Use |  | -0.2 |  | 0.1 |  | -1.4 |  | 0.177 |
| Delinquent Behavior | |  |  |  |  |  |  |  |  |  |
|  | Total SRD | |  |  |  |  |  |  |  |  |
|  |  | **Moderate Poly-inhalant Use vs. Severe Poly-inhalant Use** |  | **-9.9** |  | **0.8** |  | **-12.5** |  | **0.016** |
|  |  | **Gas & Perm. Markers Use vs. Severe Poly-inhalant Use** |  | **-9.8** |  | **0.8** |  | **-12.7** |  | **0.015** |
|  |  | **Low-Use vs. Severe Poly-inhalant Use** |  | **-13.5** |  | **0.6** |  | **-20.8** |  | **0.000** |
|  |  | Gas & Perm. Markers Use vs. Moderate Poly-inhalant Use |  | 0.1 |  | 0.8 |  | 0.1 |  | 1.000 |
|  |  | Low-Use vs. Moderate Poly-inhalant Use |  | -3.6 |  | 0.6 |  | -5.8 |  | 0.644 |
|  |  | Low-Use vs. Gas & Perm. Markers Use |  | -3.7 |  | 0.6 |  | -6.1 |  | 0.522 |
|  | SRD Property Crime Index | |  |  |  |  |  |  |  |  |
|  |  | **Moderate Poly-inhalant Use vs. Severe Poly-inhalant Use** |  | **-5.9** |  | **0.6** |  | **-9.4** |  | **0.034** |
|  |  | **Gas & Perm. Markers Use vs. Severe Poly-inhalant Use** |  | **-6.1** |  | **0.6** |  | **-10.1** |  | **0.021** |
|  |  | **Low-Use vs. Severe Poly-inhalant Use** |  | **-9.1** |  | **0.5** |  | **-18.2** |  | **0.000** |
|  |  | Gas & Perm. Markers Use vs. Moderate Poly-inhalant Use |  | -0.2 |  | 0.5 |  | -0.4 |  | 1.000 |
|  |  | Low-Use vs. Moderate Poly-inhalant Use |  | -3.3 |  | 0.4 |  | -7.6 |  | 0.132 |
|  |  | Low-Use vs. Gas & Perm. Markers Use |  | -3.0 |  | 0.4 |  | -7.7 |  | 0.166 |
|  | Age at onset of offending (years) | |  |  |  |  |  |  |  |  |
|  |  | Moderate Poly-inhalant Use vs. Severe Poly-inhalant Use |  | 0.6 |  | 0.3 |  | 2.0 |  | 1.000 |
|  |  | Gas & Perm. Markers Use vs. Severe Poly-inhalant Use |  | 0.6 |  | 0.3 |  | 1.9 |  | 1.000 |
|  |  | **Low-Use vs. Severe Poly-inhalant Use** |  | **1.2** |  | **0.2** |  | **4.9** |  | **0.025** |
|  |  | Gas & Perm. Markers Use vs. Moderate Poly-inhalant Use |  | -0.1 |  | 0.3 |  | -0.3 |  | 1.000 |
|  |  | Low-Use vs. Moderate Poly-inhalant Use |  | 0.6 |  | 0.2 |  | 2.8 |  | 0.554 |
|  |  | Low-Use vs. Gas & Perm. Markers Use |  | 0.7 |  | 0.2 |  | 3.3 |  | 0.302 |
| Brief Symptom Inventory | | |  |  |  |  |  |  |  |  |
|  | Global Severity Index | |  |  |  |  |  |  |  |  |
|  |  | **Moderate Poly-inhalant Use vs. Severe Poly-inhalant Use** |  | **-32.0** |  | **1.1** |  | **-28.3** |  | **0.000** |
|  |  | **Gas & Perm. Markers Use vs. Severe Poly-inhalant Use** |  | **-24.7** |  | **1.2** |  | **-20.2** |  | **0.000** |
|  |  | **Low-Use vs. Severe Poly-inhalant Use** |  | **-42.2** |  | **0.9** |  | **-44.8** |  | **0.000** |
|  |  | Gas & Perm. Markers Use vs. Moderate Poly-inhalant Use |  | 7.3 |  | 1.0 |  | 7.7 |  | 1.000 |
|  |  | Low-Use vs. Moderate Poly-inhalant Use |  | -10.2 |  | 0.7 |  | -14.3 |  | 0.081 |
|  |  | **Low-Use vs. Gas & Perm. Markers Use** |  | **-17.5** |  | **0.7** |  | **-24.2** |  | **0.000** |
|  | Somatization | |  |  |  |  |  |  |  |  |
|  |  | **Moderate Poly-inhalant Use vs. Severe Poly-inhalant Use** |  | **-4.1** |  | **0.4** |  | **-10.1** |  | **0.000** |
|  |  | **Gas & Perm. Markers Use vs. Severe Poly-inhalant Use** |  | **-2.7** |  | **0.4** |  | **-6.4** |  | **0.002** |
|  |  | **Low-Use vs. Severe Poly-inhalant Use** |  | **-4.1** |  | **0.4** |  | **-11.5** |  | **0.000** |
|  |  | Gas & Perm. Markers Use vs. Moderate Poly-inhalant Use |  | 1.4 |  | 0.3 |  | 4.5 |  | 0.195 |
|  |  | Low-Use vs. Moderate Poly-inhalant Use |  | -0.3 |  | 0.2 |  | -1.4 |  | 0.939 |
|  |  | **Low-Use vs. Gas & Perm. Markers Use** |  | **-1.8** |  | **0.3** |  | **-6.9** |  | **0.006** |
|  | Obsessive–Compulsive | |  |  |  |  |  |  |  |  |
|  |  | **Moderate Poly-inhalant Use vs. Severe Poly-inhalant Use** |  | **-4.5** |  | **0.4** |  | **-10.2** |  | **0.000** |
|  |  | **Gas & Perm. Markers Use vs. Severe Poly-inhalant Use** |  | **-4.5** |  | **0.4** |  | **-10.5** |  | **0.000** |
|  |  | **Low-Use vs. Severe Poly-inhalant Use** |  | **-7.0** |  | **0.6** |  | **-10.7** |  | **0.000** |
|  |  | Gas & Perm. Markers Use vs. Moderate Poly-inhalant Use |  | 0.0 |  | 0.4 |  | 0.0 |  | 1.000 |
|  |  | **Low-Use vs. Moderate Poly-inhalant Use** |  | **-2.4** |  | **0.3** |  | **-8.3** |  | **0.001** |
|  |  | **Low-Use vs. Gas & Perm. Markers Use** |  | **-2.4** |  | **0.3** |  | **-9.1** |  | **0.000** |
|  | Interpersonal Sensitivity | |  |  |  |  |  |  |  |  |
|  |  | **Moderate Poly-inhalant Use vs. Severe Poly-inhalant Use** |  | **-2.7** |  | **0.4** |  | **-7.6** |  | **0.000** |
|  |  | Gas & Perm. Markers Use vs. Severe Poly-inhalant Use |  | -1.2 |  | 0.4 |  | -3.2 |  | 0.266 |
|  |  | **Low-Use vs. Severe Poly-inhalant Use** |  | **-2.8** |  | **0.3** |  | **-9.1** |  | **0.000** |
|  |  | **Gas & Perm. Markers Use vs. Moderate Poly-inhalant Use** |  | **1.5** |  | **0.3** |  | **5.1** |  | **0.024** |
|  |  | Low-Use vs. Moderate Poly-inhalant Use |  | -0.1 |  | 0.2 |  | -0.4 |  | 1.000 |
|  |  | **Low-Use vs. Gas & Perm. Markers Use** |  | **-1.6** |  | **0.2** |  | **-6.7** |  | **0.000** |
|  | Depression | |  |  |  |  |  |  |  |  |
|  |  | **Moderate Poly-inhalant Use vs. Severe Poly-inhalant Use** |  | **-4.0** |  | **0.4** |  | **-8.8** |  | **0.000** |
|  |  | **Gas & Perm. Markers Use vs. Severe Poly-inhalant Use** |  | **-3.7** |  | **0.4** |  | **-8.4** |  | **0.000** |
|  |  | **Low-Use vs. Severe Poly-inhalant Use** |  | **-5.7** |  | **0.4** |  | **-15.4** |  | **0.000** |
|  |  | Gas & Perm. Markers Use vs. Moderate Poly-inhalant Use |  | 0.3 |  | 0.4 |  | 0.7 |  | 1.000 |
|  |  | **Low-Use vs. Moderate Poly-inhalant Use** |  | **-1.7** |  | **0.3** |  | **-6.1** |  | **0.018** |
|  |  | **Low-Use vs. Gas & Perm. Markers Use** |  | **-2.0** |  | **0.3** |  | **-7.4** |  | **0.002** |
|  | Anxiety |  |  |  |  |  |  |  |  |  |
|  |  | **Moderate Poly-inhalant Use vs. Severe Poly-inhalant Use** |  | **-4.1** |  | **0.4** |  | **-9.7** |  | **0.000** |
|  |  | **Gas & Perm. Markers Use vs. Severe Poly-inhalant Use** |  | **-3.9** |  | **0.4** |  | **-9.2** |  | **0.000** |
|  |  | **Low-Use vs. Severe Poly-inhalant Use** |  | **-5.8** |  | **0.4** |  | **-16.5** |  | **0.000** |
|  |  | Gas & Perm. Markers Use vs. Moderate Poly-inhalant Use |  | 0.2 |  | 0.4 |  | 0.7 |  | 1.000 |
|  |  | **Low-Use vs. Moderate Poly-inhalant Use** |  | **-1.7** |  | **0.3** |  | **-6.2** |  | **0.013** |
|  |  | **Low-Use vs. Gas & Perm. Markers Use** |  | **-1.9** |  | **0.3** |  | **-7.2** |  | **0.002** |
|  | Hostility |  |  |  |  |  |  |  |  |  |
|  |  | Moderate Poly-inhalant Use vs. Severe Poly-inhalant Use |  | -2.2 |  | 0.4 |  | -5.4 |  | 0.071 |
|  |  | Gas & Perm. Markers Use vs. Severe Poly-inhalant Use |  | -1.8 |  | 0.4 |  | -4.3 |  | 0.260 |
|  |  | **Low-Use vs. Severe Poly-inhalant Use** |  | **-3.4** |  | **0.3** |  | **-9.9** |  | **0.000** |
|  |  | Gas & Perm. Markers Use vs. Moderate Poly-inhalant Use |  | 0.5 |  | 0.4 |  | 1.3 |  | 1.000 |
|  |  | Low-Use vs. Moderate Poly-inhalant Use |  | -1.2 |  | 0.3 |  | -4.3 |  | 0.321 |
|  |  | **Low-Use vs. Gas & Perm. Markers Use** |  | **-1.6** |  | **0.3** |  | **-6.2** |  | **0.030** |
|  | Phobic Anxiety | |  |  |  |  |  |  |  |  |
|  |  | **Moderate Poly-inhalant Use vs. Severe Poly-inhalant Use** |  | **-2.4** |  | **0.4** |  | **-6.8** |  | **0.000** |
|  |  | **Gas & Perm. Markers Use vs. Severe Poly-inhalant Use** |  | **-1.6** |  | **0.4** |  | **-4.4** |  | **0.031** |
|  |  | **Low-Use vs. Severe Poly-inhalant Use** |  | **-2.5** |  | **0.3** |  | **-8.0** |  | **0.000** |
|  |  | Gas & Perm. Markers Use vs. Moderate Poly-inhalant Use |  | 0.8 |  | 0.3 |  | 3.0 |  | 0.622 |
|  |  | Low-Use vs. Moderate Poly-inhalant Use |  | -0.1 |  | 0.2 |  | -0.5 |  | 1.000 |
|  |  | Low-Use vs. Gas & Perm. Markers Use |  | -0.9 |  | 0.2 |  | -4.2 |  | 0.086 |
|  | Paranoid Ideation | |  |  |  |  |  |  |  |  |
|  |  | **Moderate Poly-inhalant Use vs. Severe Poly-inhalant Use** |  | **-2.5** |  | **0.4** |  | **-6.4** |  | **0.017** |
|  |  | Gas & Perm. Markers Use vs. Severe Poly-inhalant Use |  | -1.6 |  | 0.4 |  | -4.0 |  | 0.350 |
|  |  | **Low-Use vs. Severe Poly-inhalant Use** |  | **-3.5** |  | **0.3** |  | **-11.2** |  | **0.000** |
|  |  | Gas & Perm. Markers Use vs. Moderate Poly-inhalant Use |  | 0.9 |  | 0.4 |  | 2.7 |  | 1.000 |
|  |  | Low-Use vs. Moderate Poly-inhalant Use |  | -1.0 |  | 0.3 |  | -3.9 |  | 0.396 |
|  |  | **Low-Use vs. Gas & Perm. Markers Use** |  | **-2.0** |  | **0.3** |  | **-7.4** |  | **0.002** |
|  | Psychoticism | |  |  |  |  |  |  |  |  |
|  |  | **Moderate Poly-inhalant Use vs. Severe Poly-inhalant Use** |  | **-3.0** |  | **0.4** |  | **-7.7** |  | **0.000** |
|  |  | **Gas & Perm. Markers Use vs. Severe Poly-inhalant Use** |  | **-2.6** |  | **0.4** |  | **-7.0** |  | **0.001** |
|  |  | **Low-Use vs. Severe Poly-inhalant Use** |  | **-4.3** |  | **0.3** |  | **-13.6** |  | **0.000** |
|  |  | Gas & Perm. Markers Use vs. Moderate Poly-inhalant Use |  | 0.3 |  | 0.3 |  | 1.0 |  | 0.958 |
|  |  | **Low-Use vs. Moderate Poly-inhalant Use** |  | **-1.3** |  | **0.2** |  | **-5.3** |  | **0.035** |
|  |  | **Low-Use vs. Gas & Perm. Markers Use** |  | **-1.6** |  | **0.2** |  | **-6.8** |  | **0.003** |
| Psychopathic Personality Inventory | | |  |  |  |  |  |  |  |  |
|  | Total |  |  |  |  |  |  |  |  |  |
|  |  | Moderate Poly-inhalant Use vs. Severe Poly-inhalant Use |  | -1.1 |  | 0.7 |  | -1.5 |  | 1.000 |
|  |  | Gas & Perm. Markers Use vs. Severe Poly-inhalant Use |  | -2.2 |  | 0.7 |  | -3.3 |  | 1.000 |
|  |  | **Low-Use vs. Severe Poly-inhalant Use** |  | **-9.1** |  | **0.6** |  | **-16.5** |  | **0.000** |
|  |  | Gas & Perm. Markers Use vs. Moderate Poly-inhalant Use |  | -1.1 |  | 0.6 |  | -1.8 |  | 1.000 |
|  |  | **Low-Use vs. Moderate Poly-inhalant Use** |  | **-8.0** |  | **0.5** |  | **-16.7** |  | **0.000** |
|  |  | **Low-Use vs. Gas & Perm. Markers Use** |  | **-6.9** |  | **0.4** |  | **-15.9** |  | **0.000** |
|  | Machiavellian Egocentricity | |  |  |  |  |  |  |  |  |
|  |  | **Moderate Poly-inhalant Use vs. Severe Poly-inhalant Use** |  | **-2.7** |  | **0.4** |  | **-7.4** |  | **0.004** |
|  |  | Gas & Perm. Markers Use vs. Severe Poly-inhalant Use |  | -1.1 |  | 0.4 |  | -2.9 |  | 1.000 |
|  |  | **Low-Use vs. Severe Poly-inhalant Use** |  | **-2.5** |  | **0.3** |  | **-8.5** |  | **0.001** |
|  |  | Gas & Perm. Markers Use vs. Moderate Poly-inhalant Use |  | 1.7 |  | 0.3 |  | 5.0 |  | 0.100 |
|  |  | Low-Use vs. Moderate Poly-inhalant Use |  | 0.2 |  | 0.3 |  | 0.8 |  | 1.000 |
|  |  | **Low-Use vs. Gas & Perm. Markers Use** |  | **-1.5** |  | **0.3** |  | **-5.8** |  | **0.029** |
|  | Carefree Nonplanfulness | |  |  |  |  |  |  |  |  |
|  |  | Moderate Poly-inhalant Use vs. Severe Poly-inhalant Use |  | -1.0 |  | 0.4 |  | -2.6 |  | 0.950 |
|  |  | Gas & Perm. Markers Use vs. Severe Poly-inhalant Use |  | -0.9 |  | 0.4 |  | -2.4 |  | 1.000 |
|  |  | Low-Use vs. Severe Poly-inhalant Use |  | -2.6 |  | 0.3 |  | -8.5 |  | 0.000 |
|  |  | Gas & Perm. Markers Use vs. Moderate Poly-inhalant Use |  | 0.1 |  | 0.3 |  | 0.3 |  | 1.000 |
|  |  | **Low-Use vs. Moderate Poly-inhalant Use** |  | **-1.6** |  | **0.2** |  | **-6.9** |  | **0.003** |
|  |  | **Low-Use vs. Gas & Perm. Markers Use** |  | **-1.7** |  | **0.2** |  | **-7.5** |  | **0.001** |
|  |  |  |  |  |  |  |  |  |  |  |
|  | Fearlessness | |  |  |  |  |  |  |  |  |
|  |  | Moderate Poly-inhalant Use vs. Severe Poly-inhalant Use |  | -0.5 |  | 0.4 |  | -1.3 |  | 1.000 |
|  |  | Gas & Perm. Markers Use vs. Severe Poly-inhalant Use |  | 0.0 |  | 0.4 |  | 0.0 |  | 1.000 |
|  |  | **Low-Use vs. Severe Poly-inhalant Use** |  | **-3.9** |  | **0.3** |  | **-12.0** |  | **0.000** |
|  |  | Gas & Perm. Markers Use vs. Moderate Poly-inhalant Use |  | 0.5 |  | 0.4 |  | 1.5 |  | 1.000 |
|  |  | **Low-Use vs. Moderate Poly-inhalant Use** |  | **-3.4** |  | **0.3** |  | **-12.2** |  | **0.000** |
|  |  | **Low-Use vs. Gas & Perm. Markers Use** |  | **-3.9** |  | **0.3** |  | **-15.3** |  | **0.000** |
|  | Blame Externalization | |  |  |  |  |  |  |  |  |
|  |  | Moderate Poly-inhalant Use vs. Severe Poly-inhalant Use |  | -1.9 |  | 0.4 |  | -4.9 |  | 0.185 |
|  |  | Gas & Perm. Markers Use vs. Severe Poly-inhalant Use |  | -1.0 |  | 0.4 |  | -2.7 |  | 1.000 |
|  |  | **Low-Use vs. Severe Poly-inhalant Use** |  | **-2.4** |  | **0.3** |  | **-7.9** |  | **0.005** |
|  |  | Gas & Perm. Markers Use vs. Moderate Poly-inhalant Use |  | 0.9 |  | 0.3 |  | 2.5 |  | 1.000 |
|  |  | Low-Use vs. Moderate Poly-inhalant Use |  | -0.5 |  | 0.3 |  | -1.9 |  | 1.000 |
|  |  | Low-Use vs. Gas & Perm. Markers Use |  | -1.4 |  | 0.3 |  | -5.3 |  | 0.098 |
|  | Impulsive Nonconformity | |  |  |  |  |  |  |  |  |
|  |  | Moderate Poly-inhalant Use vs. Severe Poly-inhalant Use |  | -1.7 |  | 0.4 |  | -4.4 |  | 0.122 |
|  |  | Gas & Perm. Markers Use vs. Severe Poly-inhalant Use |  | -1.0 |  | 0.4 |  | -2.7 |  | 0.923 |
|  |  | **Low-Use vs. Severe Poly-inhalant Use** |  | **-3.1** |  | **0.3** |  | **-9.9** |  | **0.000** |
|  |  | Gas & Perm. Markers Use vs. Moderate Poly-inhalant Use |  | 0.7 |  | 0.3 |  | 2.0 |  | 1.000 |
|  |  | **Low-Use vs. Moderate Poly-inhalant Use** |  | **-1.4** |  | **0.3** |  | **-5.6** |  | **0.022** |
|  |  | **Low-Use vs. Gas & Perm. Markers Use** |  | **-2.1** |  | **0.2** |  | **-8.7** |  | **0.000** |
|  | Stress Immunity | |  |  |  |  |  |  |  |  |
|  |  | **Moderate Poly-inhalant Use vs. Severe Poly-inhalant Use** |  | **2.4** |  | **0.4** |  | **6.4** |  | **0.015** |
|  |  | Gas & Perm. Markers Use vs. Severe Poly-inhalant Use |  | 1.0 |  | 0.4 |  | 2.7 |  | 1.000 |
|  |  | **Low-Use vs. Severe Poly-inhalant Use** |  | **2.5** |  | **0.3** |  | **8.4** |  | **0.001** |
|  |  | Gas & Perm. Markers Use vs. Moderate Poly-inhalant Use |  | -1.4 |  | 0.3 |  | -4.1 |  | 0.244 |
|  |  | Low-Use vs. Moderate Poly-inhalant Use |  | 0.1 |  | 0.3 |  | 0.4 |  | 1.000 |
|  |  | **Low-Use vs. Gas & Perm. Markers Use** |  | **1.5** |  | **0.2** |  | **6.0** |  | **0.020** |
| Antisocial Process Screening Device | | |  |  |  |  |  |  |  |  |
|  | Total |  |  |  |  |  |  |  |  |  |
|  |  | Moderate Poly-inhalant Use vs. Severe Poly-inhalant Use |  | -1.7 |  | 0.4 |  | -4.2 |  | 0.467 |
|  |  | Gas & Perm. Markers Use vs. Severe Poly-inhalant Use |  | -0.6 |  | 0.4 |  | -1.5 |  | 1.000 |
|  |  | Low-Use vs. Severe Poly-inhalant Use |  | -3.3 |  | 0.3 |  | -9.9 |  | 0.000 |
|  |  | Gas & Perm. Markers Use vs. Moderate Poly-inhalant Use |  | 1.1 |  | 0.4 |  | 3.0 |  | 1.000 |
|  |  | Low-Use vs. Moderate Poly-inhalant Use |  | -1.6 |  | 0.3 |  | -5.9 |  | 0.088 |
|  |  | **Low-Use vs. Gas & Perm. Markers Use** |  | **-2.7** |  | **0.3** |  | **-9.6** |  | **0.000** |
|  | Callous/Unemotional Traits | |  |  |  |  |  |  |  |  |
|  |  | Moderate Poly-inhalant Use vs. Severe Poly-inhalant Use |  | -0.3 |  | 0.3 |  | -0.9 |  | 1.000 |
|  |  | Gas & Perm. Markers Use vs. Severe Poly-inhalant Use |  | 0.4 |  | 0.3 |  | 1.1 |  | 1.000 |
|  |  | Low-Use vs. Severe Poly-inhalant Use |  | -0.9 |  | 0.3 |  | -3.3 |  | 1.000 |
|  |  | Gas & Perm. Markers Use vs. Moderate Poly-inhalant Use |  | 0.6 |  | 0.3 |  | 2.3 |  | 0.274 |
|  |  | Low-Use vs. Moderate Poly-inhalant Use |  | -0.6 |  | 0.2 |  | -3.0 |  | 0.575 |
|  |  | **Low-Use vs. Gas & Perm. Markers Use** |  | **-1.3** |  | **0.2** |  | **-5.9** |  | **0.003** |
|  | Impulsivity | |  |  |  |  |  |  |  |  |
|  |  | Moderate Poly-inhalant Use vs. Severe Poly-inhalant Use |  | -0.7 |  | 0.2 |  | -3.1 |  | 0.272 |
|  |  | **Gas & Perm. Markers Use vs. Severe Poly-inhalant Use** |  | **-1.0** |  | **0.2** |  | **-4.1** |  | **0.044** |
|  |  | **Low-Use vs. Severe Poly-inhalant Use** |  | **-2.0** |  | **0.2** |  | **-10.2** |  | **0.000** |
|  |  | Gas & Perm. Markers Use vs. Moderate Poly-inhalant Use |  | -0.2 |  | 0.8 |  | -0.3 |  | 1.000 |
|  |  | **Low-Use vs. Moderate Poly-inhalant Use** |  | **-1.3** |  | **0.2** |  | **-7.3** |  | **0.000** |
|  |  | **Low-Use vs. Gas & Perm. Markers Use** |  | **-1.0** |  | **0.2** |  | **-6.1** |  | **0.000** |
| Massachusetts Youth Screening Inventory | | |  |  |  |  |  |  |  |  |
|  | Traumatic Experiences | |  |  |  |  |  |  |  |  |
|  |  | **Moderate Poly-inhalant Use vs. Severe Poly-inhalant Use** |  | **-1.1** |  | **0.2** |  | **-5.2** |  | **0.001** |
|  |  | Gas & Perm. Markers Use vs. Severe Poly-inhalant Use |  | -0.7 |  | 0.2 |  | -3.6 |  | 0.058 |
|  |  | **Low-Use vs. Severe Poly-inhalant Use** |  | **-1.3** |  | **0.2** |  | **-8.1** |  | **0.000** |
|  |  | Gas & Perm. Markers Use vs. Moderate Poly-inhalant Use |  | 0.3 |  | 0.2 |  | 1.7 |  | 1.000 |
|  |  | Low-Use vs. Moderate Poly-inhalant Use |  | -0.2 |  | 0.2 |  | -1.5 |  | 1.000 |
|  |  | **Low-Use vs. Gas & Perm. Markers Use** |  | **-0.6** |  | **0.1** |  | **-3.9** |  | **0.013** |
|  | MAYSI-Suicide Ideation | |  |  |  |  |  |  |  |  |
|  |  | **Moderate Poly-inhalant Use vs. Severe Poly-inhalant Use** |  | **-1.9** |  | **0.3** |  | **-7.2** |  | **0.000** |
|  |  | Gas & Perm. Markers Use vs. Severe Poly-inhalant Use |  | -1.0 |  | 0.3 |  | -3.8 |  | 0.059 |
|  |  | **Low-Use vs. Severe Poly-inhalant Use** |  | **-2.8** |  | **0.2** |  | **-13.1** |  | **0.000** |
|  |  | Gas & Perm. Markers Use vs. Moderate Poly-inhalant Use |  | 0.9 |  | 0.2 |  | 3.8 |  | 0.051 |
|  |  | **Low-Use vs. Moderate Poly-inhalant Use** |  | **-0.9** |  | **0.2** |  | **-4.7** |  | **0.007** |
|  |  | **Low-Use vs. Gas & Perm. Markers Use** |  | **-1.8** |  | **0.2** |  | **-9.9** |  | **0.000** |
| Substance use and related problems | | |  |  |  |  |  |  |  |  |
|  | MAYSI-Alcohol/Drug Problems | |  |  |  |  |  |  |  |  |
|  |  | Moderate Poly-inhalant Use vs. Severe Poly-inhalant Use |  | -0.9 |  | 0.2 |  | -3.8 |  | 1.000 |
|  |  | Gas & Perm. Markers Use vs. Severe Poly-inhalant Use |  | -1.5 |  | 0.3 |  | -5.8 |  | 0.336 |
|  |  | **Low-Use vs. Severe Poly-inhalant Use** |  | **-3.0** |  | **0.2** |  | **-14.3** |  | **0.000** |
|  |  | Gas & Perm. Markers Use vs. Moderate Poly-inhalant Use |  | -0.6 |  | 0.2 |  | -13.2 |  | 1.000 |
|  |  | **Low-Use vs. Moderate Poly-inhalant Use** |  | **-2.1** |  | **0.2** |  | **-11.7** |  | **0.000** |
|  |  | **Low-Use vs. Gas & Perm. Markers Use** |  | **-1.5** |  | **0.2** |  | **-7.7** |  | **0.015** |
|  | Lifetime # of drug types used | |  |  |  |  |  |  |  |  |
|  |  | Moderate Poly-inhalant Use vs. Severe Poly-inhalant Use |  | 0.1 |  | 0.3 |  | 0.5 |  | 1.000 |
|  |  | **Gas & Perm. Markers Use vs. Severe Poly-inhalant Use** |  | **-2.5** |  | **0.3** |  | **-8.2** |  | **0.000** |
|  |  | **Low-Use vs. Severe Poly-inhalant Use** |  | **-4.2** |  | **0.3** |  | **-16.5** |  | **0.000** |
|  |  | **Gas & Perm. Markers Use vs. Moderate Poly-inhalant Use** |  | **-2.6** |  | **0.2** |  | **-10.6** |  | **0.000** |
|  |  | **Low-Use vs. Moderate Poly-inhalant Use** |  | **-4.3** |  | **0.2** |  | **-23.4** |  | **0.000** |
|  |  | **Low-Use vs. Gas & Perm. Markers Use** |  | **-1.7** |  | **0.2** |  | **-8.7** |  | **0.000** |
|  | Age at onset of alcohol use | |  |  |  |  |  |  |  |  |
|  |  | Moderate Poly-inhalant Use vs. Severe Poly-inhalant Use |  | 0.0 |  | 0.2 |  | 0.0 |  | 1.000 |
|  |  | Gas & Perm. Markers Use vs. Severe Poly-inhalant Use |  | 0.0 |  | 0.3 |  | -0.1 |  | 1.000 |
|  |  | Low-Use vs. Severe Poly-inhalant Use |  | -0.2 |  | 0.3 |  | -0.7 |  | 0.004 |
|  |  | Gas & Perm. Markers Use vs. Moderate Poly-inhalant Use |  | 0.0 |  | 0.3 |  | -0.1 |  | 1.000 |
|  |  | Low-Use vs. Moderate Poly-inhalant Use |  | -0.2 |  | 0.2 |  | -0.8 |  | 0.000 |
|  |  | Low-Use vs. Gas & Perm. Markers Use |  | -0.1 |  | 0.2 |  | -0.7 |  | 0.006 |
|  | Age at onset of marijuana use | |  |  |  |  |  |  |  |  |
|  |  | Moderate Poly-inhalant Use vs. Severe Poly-inhalant Use |  | -0.4 |  | 0.3 |  | -1.3 |  | 1.000 |
|  |  | Gas & Perm. Markers Use vs. Severe Poly-inhalant Use |  | 0.7 |  | 0.3 |  | 2.5 |  | 0.547 |
|  |  | Low-Use vs. Severe Poly-inhalant Use |  | 0.8 |  | 0.2 |  | 3.6 |  | 0.078 |
|  |  | **Gas & Perm. Markers Use vs. Moderate Poly-inhalant Use** |  | **1.1** |  | **0.2** |  | **4.3** |  | **0.016** |
|  |  | **Low-Use vs. Moderate Poly-inhalant Use** |  | **1.2** |  | **0.2** |  | **6.3** |  | **0.000** |
|  |  | Low-Use vs. Gas & Perm. Markers Use |  | 0.2 |  | 0.2 |  | 0.8 |  | 1.000 |

Note. Bolded text indicates significance after applying the alpha correction.

**Table B. Post-hoc Bivariate Analyses of Categorical Variables** with Bonferroni Alpha Corrections

| **Compar. By Variable Response** | **Severe Poly-inhalant Use** | | | **Moderate Poly-inhalant Use** | | | **Gas & Perm. Markers Use** | | | **Low-Use** | | | **Total** | | | | | | **Alpha Corr.** | **Results** | | |
| --- | --- | --- | --- | --- | --- | --- | --- | --- | --- | --- | --- | --- | --- | --- | --- | --- | --- | --- | --- | --- | --- | --- |
|  | **n** | **CC** | **%** | **n** | **CC** | **%** | **n** | **CC** | **%** | **n** | **CC** | **%** | | **n** | | **CC** | | **%** |  |  |  |  |
| Sex |  |  |  |  |  |  |  |  |  |  |  |  |  | |  | |  | |  |  |  |  |
| Male v. Female | |  |  |  |  |  |  |  |  |  |  |  |  | |  | |  | |  |  |  |  |
| Female | 9 | 1.0 | 9.6 | 10 | 0 | 10.6 | 13 | 0.5 | 13.8 | 62 | 0.3 | 66.0 | 94 | | 1.7 | | 100.0 | | 0.0250 | $\chi^{2}$(3) =2.0 *p* = 0.571 | | |
| Male | 41 | 0.1 | 6.5 | 67 | 0.0 | 10.7 | 7 | 0.1 | 11.1 | 451 | 0.0 | 71.7 | 629 | | 0.3 | | 100.0 | |  |  |  |  |
| Race/Ethnicity | | | | |  |  |  |  |  |  |  |  |  | |  | |  | |  |  |  |  |
| African American vs. White | | | | | |  |  |  |  |  |  |  |  | |  | |  | |  |  |  |  |
| **African**  **American** | **2** | **11.6** | **0.8** | **2** | **20** | **0.8** | **9** | **10.2** | **3.8** | **225** | **15.1** | **94.5** | **238** | | **56.9** | | **100.0** | | **0.0417** | **$\chi^{2}$(3)^a^= 90.7 *p* = 0.000** | | |
| **White** | **39** | **6.9** | **9.8** | **62** | **12** | **15.5** | **58** | **6.1** | **14.5** | **241** | **9.0** | **60.3** | **400** | | **33.9** | | **100.0** | |  |  |  |  |
| **African American vs. Latino/Latina** | | | | | | |  |  |  |  |  |  |  | |  | |  | |  |  |  |  |
| **African**  **American** | **2** | **2.1** | **0.8** | **2** | **3.7** | **0.8** | **9** | **0.6** | **3.8** | **225** | **0.6** | **94.5** | **238** | | **7** | | **100.0** | |  | **$\chi^{2}$(3)= 366.6 *p* = 0.000** | | |
| **Latino/**  **Latina** | **4** | **18.0** | **14.3** | **6** | **32** | **21.4** | **4** | **5.1** | **14.3** | **14** | **4.9** | **50.0** | **28** | | **59.6** | | **100.0** | |  |  |  |  |
| **African American vs. Biracial** | | | | |  |  |  |  |  |  |  |  |  | |  | |  | |  |  |  |  |
| **African**  **American** | **2** | **1.2** | **0.8** | **2** | **4.1** | **0.8** | **9** | **1.0** | **3.8** | **225** | **0.6** | **94.5** | **238** | | **6.9** | | **100.0** | |  | **$\chi^{2}$(3)= 43.4 *p* = 0.000** | | |
| **Biracial** | **3** | **6.1** | **6.7** | **7** | **22** | **15.6** | **6** | **5.5** | **13.3** | **29** | **3.2** | **64.4** | **45** | | **36.5** | | **100.0** | |  |  |  |  |
| White vs. Latino/Latina | | | | | | |  |  |  |  |  |  |  | |  | |  | |  |  |  |  |
| White | 39 | 0.0 | 9.8 | 62 | 0.0 | 15.5 | 58 | 0.0 | 14.5 | 241 | 0.0 | 60.3 | 400 | | 0.1 | | 100.0 | |  | $\chi^{2}$(3)= 1.6 *p* = 0.552 | | |
| Latino/  Latina | 4 | 0.5 | 14.3 | 6 | 0.5 | 21.4 | 4 | 0.0 | 14.3 | 14 | 0.4 | 50.0 | 28 | | 1.5 | | 100.0 | |  |  |  |  |
| White vs. Biracial | | | | | |  |  |  |  |  |  |  |  | |  | |  | |  |  |  |  |
| White | 39 | 0.0 | 9.8 | 62 | 0.0 | 15.5 | 58 | 0.0 | 14.5 | 241 | 0.0 | 60.3 | 400 | | 0.1 | | 100.0 | |  | $\chi^{2}$(3) = 0.563 *p* = 0.963 | | |
| Biracial | 3 | 0.4 | 6.7 | 7 | 0 | 15.6 | 6 | 0.0 | 13.3 | 29 | 0.1 | 64.4 | 45 | | 0.5 | | 100.0 | |  |  |  |  |
| Latino/Latina vs. Biracial | | | | | |  |  |  |  |  |  |  |  | |  | |  | |  |  |  |  |
| Latino/  Latina | 4 | 0.6 | 14.3 | 6 | 0.2 | 21.4 | 4 | 0.0 | 14.3 | 14 | 0.4 | 50.0 | 28 | | 1.2 | | 100.0 | |  | $\chi^{2}$(3) = 2.0 *p* = 0.580 | | |
| Biracial | 3 | 0.4 | 6.7 | 7 | 0.1 | 15.6 | 6 | 0.0 | 13.3 | 29 | 0.2 | 64.4 | 45 | | 0.8 | | 100.0 | |  |  |  |  |
| Geographic Area | | | | | |  |  |  |  |  |  |  |  | |  | |  | |  |  |  |  |
| Urban City vs. Suburban Area | | | | | |  |  |  |  |  |  |  |  | |  | |  | |  |  |  |  |
| Urban  City | 18 | 0.1 | 6.4 | 18 | 0.1 | 6.4 | 18 | 0.5 | 6.4 | 229 | 0.2 | 80.9 | 283 | | 0.9 | | 100.0 | | 0.042 | $\chi^{2}$(3) = 3.3 *p* = 0.351 | | |
| Suburban  Area | 8 | 0.2 | 8.0 | 8 | 0.2 | 8.0 | 11 | 1.6 | 11.0 | 73 | 0.4 | 73 | 100 | | 2.4 | | 100.0 | |  |  |  |  |
| **Urban City vs. Small Town** | | | | |  |  |  |  |  |  |  |  |  | |  | |  | |  |  |  |  |
| **Urban**  **City** | **18** | **0.0** | **6.4** | **18** | **6.0** | **6.4** | **18** | **5.7** | **6.4** | **229** | **3.9** | **80.9** | **283** | | **15.6** | | **100.0** | |  | **$\chi^{2}$(3) = 31.1 *p* = 0.000** | | |
| **Small**  **Town** | **20** | **0.0** | **7.0** | **46** | **5.9** | **16.1** | **45** | **5.6** | **15.7** | **175** | **3.9** | **61.2** | **286** | | **15.5** | | **100.0** | |  |  |  |  |
| Urban City vs. Rural or Country Area | | | | | | |  |  |  |  |  |  |  | |  | |  | |  |  |  |  |
| Urban  City | 18 | 0.0 | 6.4 | 18 | 0.1 | 6.4 | 18 | 0.5 | 6.4 | 229 | 0.2 | 80.9 | 283 | | 0.9 | | 100.0 | |  | $\chi^{2}$(3) = 7.8 *p* = 0.049 | | |
| Rural or  Country Area | 4 | 0.1 | 7.4 | 5 | 0.5 | 9.3 | 9 | 5.0 | 16.7 | 36 | 1.0 | 66.7 | 54 | | 6.6 | | 100.0 | |  |  |  |  |
| Suburban Area vs. Small Town | | | | | |  |  |  |  |  |  |  |  | |  | |  | |  |  |  |  |
| Suburban  Area | 8 | 0.1 | 8.0 | 8 | 2.6 | 8.0 | 11 | 0.8 | 11.0 | 73 | 1.2 | 73 | 100 | | 4.7 | | 100.0 | |  | $\chi^{2}$(3) = 6.3 *p* = 0.097 | | |
| Small  Town | 20 | 0.0 | 7.0 | 46 | 0.9 | 16.1 | 45 | 0.3 | 15.7 | 175 | 0.4 | 61.2 | 286 | | 1.6 | | 100.0 | |  |  |  |  |
| Suburban Area vs. Rural or Country Area | | | | | |  |  |  |  |  |  |  |  | |  | |  | |  |  |  |  |
| Suburban  Area | 8 | 0.0 | 8.0 | 8 | 0.0 | 8.0 | 11 | 0.3 | 11.0 | 73 | 0.1 | 73 | 100 | | 0.4 | | 100.0 | |  | $\chi^{2}$(3) = 1.1 *p* = 0.756 | | |
| Rural or  Country Area | 4 | 0.0 | 7.4 | 5 | 0.0 | 9.3 | 9 | 0.6 | 16.7 | 36 | 0.1 | 66.7 | 54 | | 0.7 | | 100.0 | |  |  |  |  |
| Small Town vs. Rural or Country Area | | | | | |  |  |  |  |  |  |  |  | |  | |  | |  |  |  |  |
| Small  Town | 20 | 0.0 | 7.0 | 46 | 0.2 | 16.1 | 45 | 0.0 | 15.7 | 175 | 0.0 | 61.2 | 286 | | 0.3 | | 100.0 | |  | $\chi^{2}$(3) = 1.7 *p* = 0.646 | | |
| Rural or  Country  Area | 4 | 0.0 | 7.4 | 5 | 1.2 | 9.3 | 9 | 0.0 | 16.7 | 36 | 0.2 | 66.7 | 54 | | 1.4 | | 100.0 | |  |  |  |  |
| Receives Welfare | | | |  |  |  |  |  |  |  |  |  |  | |  | |  | |  |  |  |  |
| Yes v. No |  |  |  |  |  |  |  |  |  |  |  |  |  | |  | |  | |  |  |  |  |
| Yes | 18 | 0.2 | 6.3 | 29 | 0.1 | 10.1 | 37 | 0.5 | 12.9 | 204 | 0.0 | 70.8 | 426 | | 0.6 | | 100.0 | | 0.0250 | $\chi^{2}$(3) = 1.4 *p* = 0.705 | | |
| No | 32 | 0.2 | 7.5 | 48 | 0.1 | 11.3 | 45 | 0.3 | 10.6 | 301 | 0.0 | 70.7 | 288 | | 0.8 | | 100.0 | |  |  |  |  |
| **Head Injury Caused Blackout** | | | | |  |  |  |  |  |  |  |  |  | |  | |  | |  |  |  |  |
| **Yes v. No** |  |  |  |  |  |  |  |  |  |  |  |  |  | |  | |  | |  |  |  |  |
| **Yes** | **17** | **6.7** | **12.9** | **18** | **1.1** | **13.6** | **18** | **0.5** | **13.6** | **79** | **2.2** | **59.9** | **132** | | **10.5** | | **100.0** | | **0.0250** | **$\chi^{2}$(3) = 12.9 *p* = 0.006** | | |
| **No** | **33** | **1.5** | **5.6** | **59** | **0.2** | **10.0** | **65** | **0.1** | **11.1** | **431** | **0.5** | **73.3** | **588** | | **2.4** | | **100.0** | |  |  |  |  |
| **Diagnosed With Mental Illness** | | | | | |  |  |  |  |  |  |  |  | |  | |  | |  |  |  |  |
| **Yes v. No** |  |  |  |  |  |  |  |  |  |  |  |  |  | |  | |  | |  |  |  |  |
| **Yes** | **42** | **11.2** | **11.4** | **51** | **3.3** | **13.8** | **55** | **3.6** | **14.9** | **222** | **6.3** | **60.0** | **370** | | **24.4** | | **100.0** | | **0.0250** | **$\chi^{2}$(3) = 50.2 *p* = 0.000** | | |
| **No** | **7** | **11.9** | **2.0** | **26** | **3.5** | **7.4** | **28** | **3.8** | **8** | **289** | **6.6** | **82.6** | **350** | | **25.8** | | **100.0** | |  |  |  |  |
| **Heard Voices of People Not There** | | | | | |  |  |  |  |  |  |  |  | |  | |  | |  |  |  |  |
| **Yes v. No** |  |  |  |  |  |  |  |  |  |  |  |  |  | |  | |  | |  |  |  |  |
| **Yes** | **15** | **8.0** | **14.2** | **11** | **0.0** | **10.4** | **25** | **13.5** | **23.6** | **55** | **5.4** | **51. 9** | **106** | | **27.0** | | **100.0** | | **0.0250** | **$\chi^{2}$(3) = 31.6 *p* = 0.000** | | |
| **No** | **35** | **1.4** | **5.7** | **66** | **0.0** | **10.0** | **58** | **2.3** | **9.4** | **458** | **0.9** | **74.2** | **617** | | **4.6** | | **100.0** | |  |  |  |  |
| **Lifetime Alcohol Use** | | | | |  |  |  |  |  |  |  |  |  | |  | |  | |  |  |  |  |
| **Yes v. No** |  |  |  |  |  |  |  |  |  |  |  |  |  | |  | |  | |  |  |  |  |
| **Yes** | **15** | **8.0** | **14.2** | **11** | **0.0** | **10.4** | **25** | **13.5** | **23.6** | **55** | **5.4** | **51. 9** | **106** | | **27.0** | | **100.0** | | **0.0250** | **$\chi^{2}$(3) = 31.6 *p* = 0.000** | | |
| **No** | **35** | **1.4** | **5.7** | **66** | **0.0** | **10.7** | **58** | **2.3** | **9.4** | **458** | **0.9** | **74.2** | **617** | | **4.6** | | **100.0** | |  |  |  |  |
| **Lifetime Marijuana Use** | | | | | |  |  |  |  |  |  |  |  | |  | |  | |  |  |  |  |
| **Yes v. No** |  |  |  |  |  |  |  |  |  |  |  |  |  | |  | |  | |  |  |  |  |
| **Yes** | **47** | **0.3** | **7.5** | **77** | **1.6** | **12.3** | **76** | **0.2** | **12.1** | **426** | **0.7** | **68.1** | **626** | | **2.9** | | **100.0** | | **0.0250** | **$\chi^{2}$(3) = 21.6 *p* = 0.000** | | |
| **No** | **3** | **2.0** | **3.1** | **0** | **10** | **0.0** | **7** | **1.5** | **7.2** | **87** | **4.8** | **89.7** | **97** | | **18.7** | | **100.0** | |  |  |  |  |

Note. CC = Chi-square contribution; Alpha Corr. = Bonferroni Alpha Correction. Bolded text indicates significance after applying the alpha correction.

^a^ When cell sizes were below 5 in more than 20% of the cells the likelihood-ratio chi-square test was used.
